# Terrestrial land-cover type richness is positively linked to landscape-level functioning

**DOI:** 10.1038/s41467-019-14002-7

**Published:** 2020-01-09

**Authors:** Jacqueline Oehri, Bernhard Schmid, Gabriela Schaepman-Strub, Pascal A. Niklaus

**Affiliations:** 1 Department of Evolutionary Biology and Environmental Studies, University of Zurich, Winterthurerstrasse 190, CH-8057 Zurich, Switzerland; 2 Department of Geography, University of Zurich, Winterthurerstrasse 190, CH-8057 Zurich, Switzerland

**Keywords:** Biodiversity, Community ecology, Ecosystem ecology, Macroecology

## Abstract

Biodiversity–ecosystem functioning (BEF) experiments have shown that local species richness promotes ecosystem functioning and stability. Whether this also applies under real-world conditions is still debated. Here, we focus on larger scales of space, time and ecological organization. We develop a quasi-experimental design in which we relate land-cover type richness as measure of landscape richness to 17-year time series of satellite-sensed functioning in 4974 landscape plots 6.25 or 25 ha in size. We choose plots so that landscape richness is orthogonal to land cover-type composition and environmental conditions across climatic gradients. Landscape-scale productivity and temporal stability increase with landscape richness, irrespective of landscape plot size. Peak season near-infrared surface albedo, which is relevant for surface energy budgets, is higher in mixed than in single land-cover type landscapes. Effect sizes are as large as those reported from BEF-experiments, suggesting that landscape richness promotes landscape functioning at spatial scales relevant for management.

## Abstract (web summary)

Species richness is often reported to enhance ecosystem functioning, but it is unclear whether similar diversity-functioning relationships occur at larger scales. Here Oehri et al. combine land cover survey and remote sensing data to show a positive relationship between landscape diversity and landscape functioning.

## Introduction

A large body of evidence supports the notion of positive biodiversity effects on ecosystem functioning^1^. This evidence mainly originates from studies in which plot-level species richness was manipulated experimentally and diversity effects were assessed for selected ecosystem functions, with productivity being the most prominent one. With few exceptions, the broad pattern emerging is one of positive decelerating responses of productivity, i.e. productivity increases with species richness but the incremental benefit of adding species is largest at low diversity^1^. A second important general trend is that species-rich ecosystems buffer disturbances more effectively than species-poor ecosystems, resulting in a higher temporal stability of ecosystem functioning^1,2^. However, BEF studies to date have mostly focused on species-level richness and relatively small experimental field plots^3–5^. These plots typically harbor a single plant community only and treatment randomization statistically decouples species richness from the structure and environmental conditions in the surrounding of these plots. This markedly contrasts real-world landscapes^6–8^, which resemble a heterogeneous patchwork of land-cover types, including cultural and natural elements^9,10^. Different land-cover types assemble to a complex landscape with small and large patches that are intertwined to various degrees^11,12^.

Phenomenologically, a positive diversity effect indicates that systems composed of multiple different units (often called “mixtures”) in general perform better than systems composed of fewer different or identical units (often called “monocultures”). Positive BEF effects thus occur when the average interaction among non-equal units is more positive with respect to the consequences for system functioning than the average interaction among equal units. To the best of our knowledge, so far BEF research has exclusively focused on groups of individuals, mostly plants, as basic units of diversity. These units have been defined based on taxonomic, phylogenetic, or functional attributes of individuals, for example species identity^1,13^, genotype^14,15^, growth form^16^, or metabolic capabilities such as the one to engage in symbiotic associations allowing fixation of atmospheric dinitrogen^17,18^. However, it is evident that interactions that shape the functioning of systems can also occur at other organizational levels. For example, heterozygous and polyploid organisms typically show enhanced performance, which is at least in part due to intra-individual genetic diversity effects, i.e. due to a biodiversity effect at a lower level of organization^19^. An alternative perspective, which is the one we introduce here, is that diversity effects could also occur with respect to the richness of larger organizational units, namely of entire land-cover type units, in brief “land-cover units”. Land-cover type richness, in brief “landscape richness”, encompasses mostly differences among ecosystems but also includes largely abiotic land-cover types such as urban areas. In real-world landscapes, land-cover units are interconnected through the exchange of energy, matter, and organisms^9,11,12,20^. Most research in interconnected ecosystems (which sometimes are referred to as “meta-ecosystems”^20^) has been concerned with the dispersal of species across networks of relatively similar land-cover units^21^. However, there is evidence for a wide range of additional, functionally important interactions among interconnected land-cover units. For example, resources such as organic carbon and nutrients are moved between land-cover units. The transport of these resources occurs in part passively (e.g. by wind or gravity) but often also is mediated by animals that forage and defecate in different land-cover units, thereby re-locating nutrients, or that migrate in the course of their life cycle and end up as carcasses depositing resources that were originally assimilated in different places^12^. These processes are different from dispersal, and there is growing evidence that such resource transport rates between different land-cover units often are large and shape local ecosystem functions^22,23^. Finally, significant amounts of water and energy are moved between land-cover units through atmospheric processes. Differences in surface energy budgets drive heat-island and oasis effects that depend on size and spatial configuration of land-cover units^24,25^. These island effects can reach hundreds of meters or even kilometers into adjacent land-cover units^26^. Surface roughness and resulting wind shear affect spatial transport of heat at scales beyond the ones of individual land-cover units^27^. Overall, it thus is evident that numerous spatial links exist among land-cover units and that these support novel emergent interactions in real-world landscapes^11,28^. However, we only begin to understand their functional consequences^22,24^ and virtually nothing is known about whether these sum up to relevant diversity effects at the landscape scale.

**Fig. 1:**
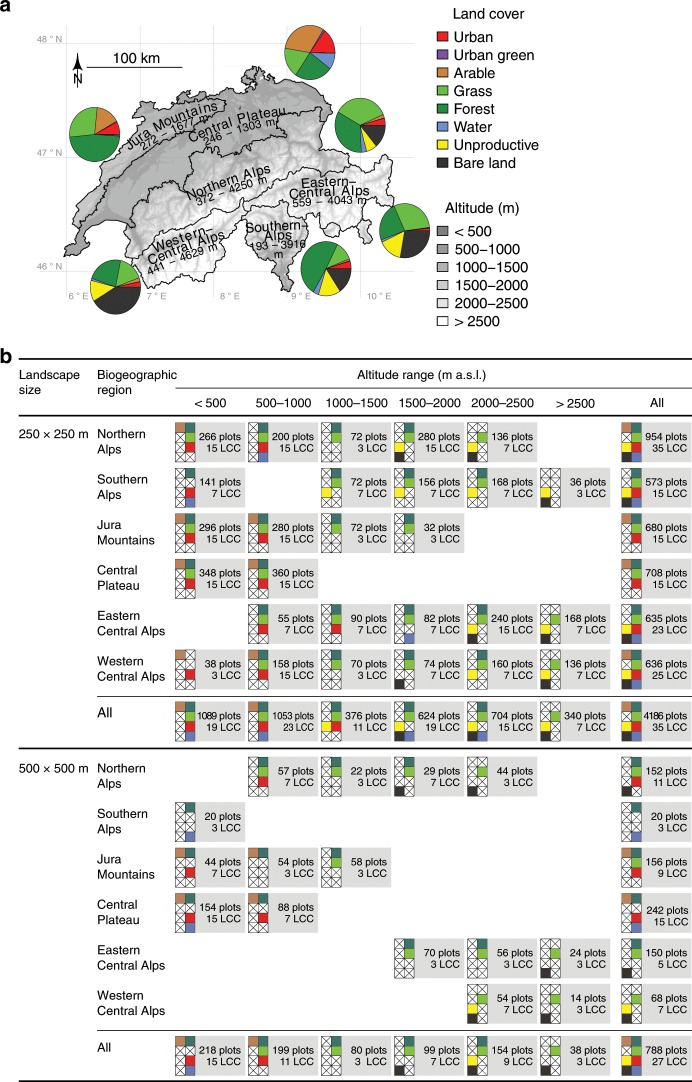
Study design: study area and selected networks of 250 × 250 m and 500 × 500 m landscapes. **a** The study area was divided into blocks defined by biogeographic region (BGR; names in map) and altitude ranges. Pie charts show the fractional cover of the land-cover types in the different BGRs. **b** Within each block, the largest possible set of land-cover types was chosen that allowed constructing gradients in landscape richness with all possible land-cover combinations realized (see (a) for color legend; crosses indicate land-cover types not used in the respective block). Landscape richness was thus orthogonal to fractional contribution of any land-cover type. Plots: the number of landscape plots; LCC: the number of unique land-cover compositions in the corresponding BGR and altitude ranges.

Here, we adopt a landscape-level system’s perspective^11^ to test whether the richness of large ecological units, namely of land-cover types, affects the functioning and stability of landscapes. In other words, we use land-cover type richness (short “landscape richness”) as a measure of landscape diversity. With this shift in spatial and organizational scale from local species richness (α-diversity of species) and ecosystem functioning in plots^1^ to landscape richness (α-diversity of land-cover types) and landscape functioning we allow for the detection of landscape richness–landscape functioning relationships that are mediated through larger scale interactions among land-cover units as discussed above. Our approach is phenomenological, i.e. we focus on the statistical relationship between landscape richness and landscape functioning rather than on specific processes. This is not unlike most plot-scale BEF research which has revealed robust relationships between species richness and ecosystem functioning whereas the underlying specific mechanisms remained surprisingly enigmatic^29,30^ except for few cases^18^. Specifically, we measure landscape functioning in terms of primary productivity, vegetation phenology, land-surface albedo, and the temporal stability of these variables. We derive these variables from satellite-sensed data available at high spatial resolution (MODIS EVI and albedo products^31,32^, years 2000–2016). A notorious challenge in observational studies is that correlated drivers cannot be separated statistically. To maximize statistical power and minimize such confounding in inferred cause–effect relationships, we use design principles from experimental BEF research^33^. We first divide our study area into blocks based on the combination of six biogeographic regions covered in our study area^34^ and six altitude intervals partitioning the total elevation range (193–3279 m a.s.l.; Fig. 1). The rationale underlying these blocks is to account for variation across the study area with respect to the pool of land-cover types present in each block, environmental conditions and landscape management (see Fig. 1; and references^34,35^). Within each block, we select landscapes that span land-cover type richness gradients that are orthogonal to land-cover type composition; in other words, the fractional contribution of each land-cover type remains constant across the landscape richness gradient (Fig. 1b). The landscapes within a block are selected randomly, with the constraint that they need to be well-spread in space, land-cover type evenness needs to be high in mixed land-cover type landscapes, and that altitude, slope angle, and the north-south aspect of the slope are distributed similarly across all land-cover type compositions. This procedure is repeated separately for landscape plots of 250 × 250 m and 500 × 500 m area, resulting in two independent, non-overlapping data sets of 4186 and 788 landscapes, respectively.

Our study demonstrates that landscape-level vegetation functioning and its temporal stability increase with landscape richness, most likely through species richness-independent mechanisms that emerge at these large scales.

## Results

### Primary productivity and phenology

**Fig. 2:**
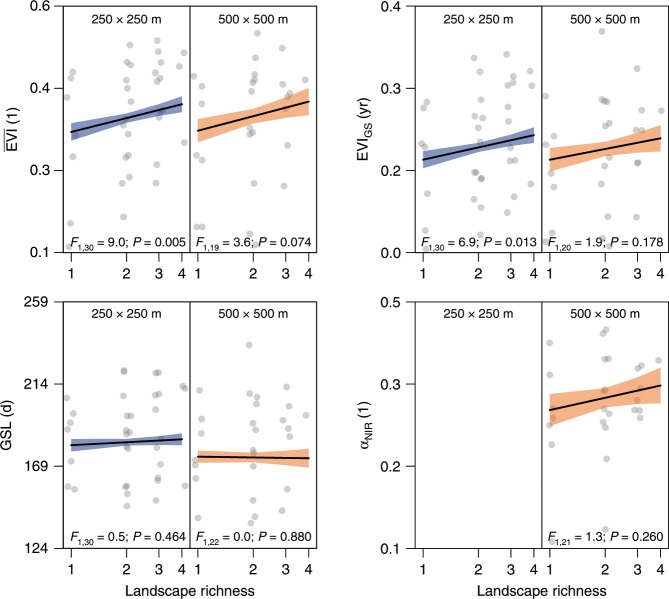
Effects of landscape richness on landscape functioning variables. Productivity: growing-season mean enhanced vegetation index ($\overline {{\mathrm{EVI}}}$), growing-season integrated enhanced vegetation index (EVI_GS_); Phenology: growing-season length (GSL); Albedo: near-infrared range (α_NIR_, 500 × 500 m landscape plots only); black line and shaded areas: model-predicted mean ± s.e.m.; *F*: *F*-ratio with associated degrees of freedom; *P*: *P*-value. Symbols indicate averages for each land-cover composition. Note the log-transformed axis for landscape richness (*n* = 237 and 77 for 250 × 250 m and 500 × 500 m land-cover composition × block combinations, respectively). Source data are provided as a Source Data file.

We used a satellite-sensed enhanced vegetation index (EVI) as measure of primary productivity. Average growing-season productivity ($\overline {{\mathrm{EVI}}}$) and integrated growing-season productivity (EVI_GS_; see Methods and ref. ^35^) increased significantly with log-transformed landscape richness [log(LR)] in 250 × 250 m plots (*F*_1,30_ = 9.0, *P* = 0.005) and trend-wise in 500 × 500 m plots (*F*_1,19_ = 3.6, *P* = 0.074; Fig. 2). The log(LR) effects were similar for annual peak EVI (EVI_max_; 250 × 250 m: *F*_1,30_ = 7.6, *P* = 0.010; 500 × 500 m: *F*_1,16_ = 3.7, *P* = 0.071). The net diversity effect (NE^36^) was positive for both $\overline {{\mathrm{EVI}}}$ and EVI_GS_ (*P* = 0.001–0.003 depending on landscape size; Table 1), i.e. both $\overline {{\mathrm{EVI}}}$ and EVI_GS_ “overyielded”^37,38^ in mixed landscapes relative to the average of the corresponding single land-cover type landscapes. Within mixtures, the net diversity effect (NE) increased significantly with log(LR) in 250 × 250 m plots, with a similar but less significant trend in 500 × 500 m plots (Table 1). Growing-season length (GSL; Methods; ref. ^35^) did not depend on log(LR) (Table 1; Fig. 2). The effects of log(LR) were consistent across blocks [block × log(LR): n.s.].

**Table 1:** Net diversity effects (NE) on productivity, phenology and albedo.

Category	Landscape function	Landscape size	NE > 0 effect (mean ± s.e.m.)^a^	NE > 0 effect significance (one-sample *t*-test)^a^	Linear effect of log(LR) on NE (mean ± s.e.m.)^a^	Linear effect of log(LR) on NE significance (*F*-test)^a^
Productivity	$\overline {{\mathrm{EVI}}}$	250 × 250 m	**0.026** **±** **0.008**	***t***_**27**_ = **3.3;** ***P*** =** 0.001**	**0.054** **±** **0.019**	***F***_**1,20**_ = **7.7;** ***P*** = **0.012**
		500 × 500 m	**0.035** **±** **0.011**	***t***_**19**_ = **3.2;** ***P*** = **0.003**	0.087 ± 0.045	*F*_1,16_ = 3.8; *P* = 0.067
	EVI_GS_ (yr)	250 × 250 m	**0.017** **±** **0.005**	***t***_**27**_ = **3.6;** ***P*** = **0.001**	**0.033** **±** **0.013**	***F***_**1,18**_ = **6.8;** ***P*** = **0.018**
		500 × 500 m	**0.022** **±** **0.006**	***t***_**19**_ = **3.7;** ***P*** = **0.001**	0.045 ± 0.025	*F*_1,15_ = 3.3; *P* = 0.088
Phenology	GSL (d)	250 × 250 m	1.807 ± 1.346	*t*_27_ = 1.3; *P* = 0.095	3.364 ± 3.561	*F*_1,19_ = 0.9; *P* = 0.357
		500 × 500 m	0.295 ± 1.862	*t*_19_ = 0.2; *P* = 0.438	−2.101 ± 8.644	*F*_1,24 = _0.1; *P* = 0.810
Albedo	α_NIR_	500 × 500 m	**0.030** **±** **0.007**	***t***_**19**_ = **4.2;** ***P*** <** 0.001**	0.055 ± 0.028	*F*_1,17_ = 3.9; *P* = 0.064

^a^Statistically significant results are highlighted in bold

The first test (NE > 0) indicates whether landscape plots with a mixture of land-cover types performed better than landscape plots with a single land-cover type. The second test [linear effect of log(LR)] indicates whether NE increased with log-transformed landscape richness [log(LR)]. Productivity: growing-season mean enhanced vegetation index ($\overline {{\mathrm{EVI}}}$), growing-season integrated enhanced vegetation index (EVI_GS_); Phenology: growing-season length (GSL); Albedo: near-infrared range (α_NIR_); *t*: *t*-value with associated degrees of freedom; *F*: *F*-ratio with associated degrees of freedom; *P*: *P*-value (*n* = 153 and 40 land-cover composition × block combinations with landscape richness > 1 in 250 × 250 m and 500 × 500 m landscapes, respectively)

### Land surface albedo

Summer near-infrared albedo (α_NIR;_ June–August) increased with log(LR). Because the land-cover types differed substantially in albedo, this effect was statistically significant only for the net diversity effect NE, which adjusts for this difference in baseline values (Methods; Table 1). Within mixtures, NE increased with landscape richness [Table 1; *P* = 0.049 for LR; *P* = 0.064 for log(LR)]. This effect was found in all blocks but differed in magnitude depending on altitude. No effects of log(LR) were found for albedo in the visible (α_vis_) or total short-wave (α_SW_) range.

### Temporal stability

**Fig. 3:**
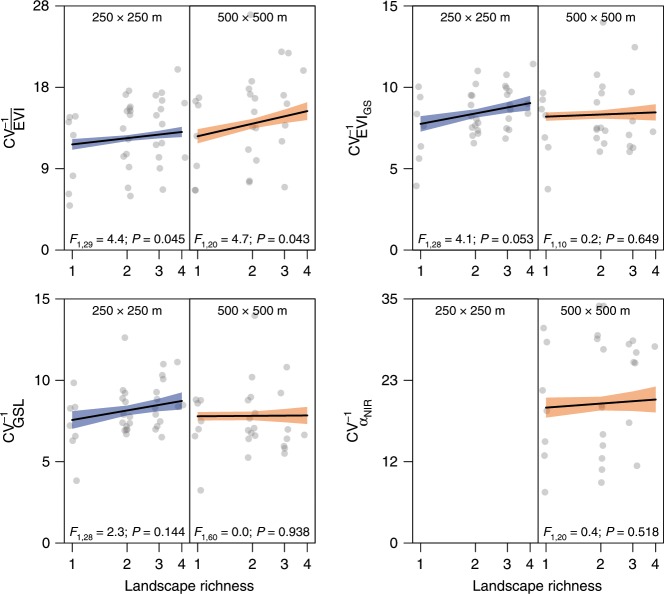
Effects of landscape richness on the temporal stability of landscape functioning variables. Stability is expressed as inverse coefficient of variation (CV^−1^). Productivity: growing-season mean enhanced vegetation index ($\overline {{\mathrm{EVI}}}$), growing-season integrated enhanced vegetation index (EVI_GS_). Phenology: growing-season length (GSL); Albedo: near-infrared range (α_NIR_, 500 × 500 m landscape plots only); black line and shaded areas: model-predicted mean ± s.e.m.; *F*: *F*-ratio with associated degrees of freedom; *P*: *P*-value. Symbols indicate averages for each land-cover composition (*n* = 237 and 77 for 250 × 250 m and 500 × 500 m land-cover composition × block combinations, respectively). Source data are provided as a Source Data file.

We used the inverse coefficient of inter-annual variation (CV^−1^; years 2000–2016) as measure of stability. In 250 × 250 m plots, log(LR) promoted the stability of average growing-season productivity (${\mathrm{CV}}_{\overline {{\mathrm{EVI}}} }^{ - 1}$; F_1,29_ = 4.4, *P* = 0.045), integrated growing-season productivity (${\mathrm{CV}}_{{\mathrm{EVI}}_{{\mathrm{GS}}}}^{ - 1}$; *F*_1,28_ = 4.1, *P* = 0.053), and maximum growing-season productivity (${\mathrm{CV}}_{{\mathrm{EVI}}_{{\mathrm{max}}}}^{ - 1}$; *F*_1,29_ = 5.8, *P* = 0.022). Similar but weaker patterns were found in 500 × 500 m plots, where effects were significant for ${\mathrm{CV}}_{\overline {{\mathrm{EVI}}} }^{ - 1}$ (*F*_1,20_ = 4.7, *P* = 0.043; Fig. 3). We did not detect effects of log(LR) on inter-annual stability of growing-season length (${\mathrm{CV}}_{{\mathrm{GSL}}}^{ - 1}$; Fig. 3), land-surface albedo in the near-infrared (${\mathrm{CV}}_{\alpha _{{\mathrm{NIR}}}}^{ - 1}$; Fig. 3), visible (${\mathrm{CV}}_{\alpha _{{\mathrm{vis}}}}^{ - 1}$) or total short-wave range (${\mathrm{CV}}_{\alpha _{{\mathrm{SW}}}}^{ - 1}$).

### Scale-dependence

The landscape richness–landscape functioning relationships we found were consistent between 250 × 250 m and 500 × 500 m plot sizes at which we assessed landscapes. Specifically, the estimated coefficients for the landscape richness term were statistically indistinguishable between the two data sets for small and large plots [log(LR) × scale; *P* > 0.2 for all dependent variables in general linear models combining the two data sets].

### Relative importance of landscape richness effects

**Fig. 4:**
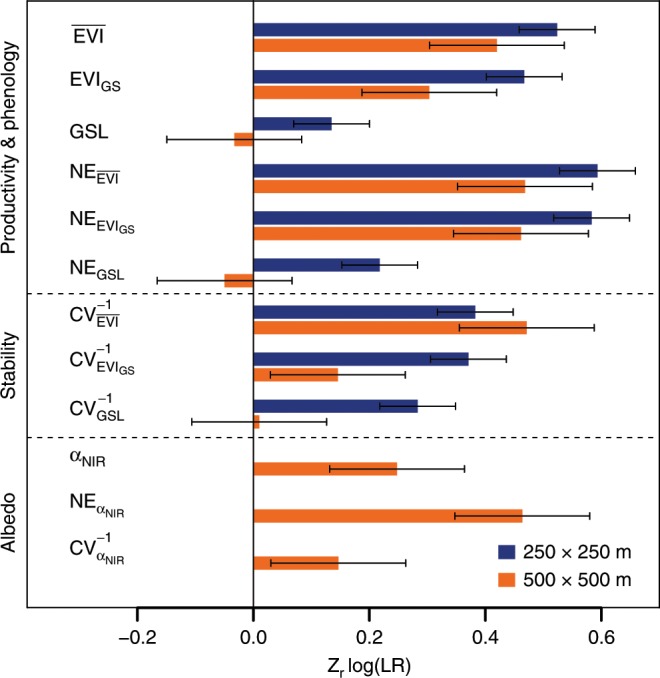
Normalized effect sizes (*Z*_r_) of landscape richness for all landscape functioning variables. log(LR): log-transformed landscape richness; $\overline {{\mathrm{EVI}}}$: growing-season mean enhanced vegetation index; EVI_GS_: growing-season integrated enhanced vegetation index; GSL: growing-season length; α_NIR_: near-infrared albedo. NE: net diversity effects for these variables; CV^−1^: temporal stability of these variables; bars show mean ± s.d. of *Z*_r_ values; blue: 250 × 250 m landscapes (*n* = 237 land-cover composition × block combinations); orange: 500 × 500 m landscapes (*n* = 77 land-cover composition × block combinations). Source data are provided as a Source Data file.

We quantified the relative importance of log(LR) for all the dependent variables analyzed by calculating normalized effect sizes (*Z*_r_; Fisher’s z-transformation based on correlation coefficients derived from *F*-ratios^39^; Fig. 4). *Z*_r_ values for log(LR) ranged from 0.30 to 0.52 for the productivity measures $\overline {{\mathrm{EVI}}}$ and EVI_GS_ and from 0.15 to 0.47 for the corresponding stability measures (${\mathrm{CV}}_{\overline {{\mathrm{EVI}}} }^{ - 1}$ and ${\mathrm{CV}}_{{\mathrm{EVI}}_{{\mathrm{GS}}}}^{ - 1}$), with no or only little differences between 250 × 250 m and 500 × 500 m landscape plots (Fig. 4). These *Z*_r_ values are comparable to the ones reported for species richness effects on ecosystem functioning in grassland BEF experiments (Methods; *Z*_r_ = 0.33 and 0.53 for effects on productivity and temporal stability of productivity, respectively).

## Discussion

In our study, we adopted design principles from experimental BEF research to test whether landscape diversity is related to landscape functioning of real-world^7^ landscapes. Expanding concepts of classical BEF experiments, we focused on larger scales of space and ecological organization and used the α-diversity of land-cover types in landscapes as measure of landscape diversity. This metric essentially captures the diversity of ecosystem types. Using a 17-year time series encompassing 4974 landscape plots deliberately selected in a quasi-experimental design^40^ to represent landscape richness gradients orthogonal to land-cover type composition, we demonstrate an increase in landscape functioning with landscape richness for satellite-sensed primary productivity, inter-annual stability of productivity, and to a lesser extent near-infrared albedo. These positive landscape richness–landscape functioning relationships were robust across broad ranges of environmental conditions defined by altitude and biogeographic region and across two plot sizes of 6.25 and 25 ha.

Biodiversity effects on the functioning of real-world ecosystems have so far mostly been analyzed with respect to local (α) species richness. These studies covered a wide range of ecosystems including drylands^41^, grasslands^42^, forests^43^ and aquatic systems^42^ and support the notion of positive BEF relationships similar to the ones observed in BEF experiments using artificially-established plots with areas in the square-meter range. Effects of spatial turnover of species, i.e. of β-diversity^44^, have received less attention. Analyses at different levels of spatial aggregation of data from spatially contiguous^45,46^ or disconnected^47,48^ natural and experimental plots suggest that the β-diversity of species also affects ecosystem functioning, in particular landscape-scale multi-functionality (but see ref. ^49^). A recent study has integrated local richness and spatial turnover of species and demonstrated positive effects of γ-species diversity on landscape-level productivity, stability and shifts in phenology in response to decadal warming^35^.

Landscape richness, the unit of diversity we use here, likely is linked to measures of β- and γ-diversity of species because ecosystems representing different land-cover types likely harbor dissimilar sets of species. However, these diversity metrics remain conceptually distinct. An interesting aspect of using larger ecological entities as basic units of diversity is that it allows integrating interactions between different ecosystem types and also with land-cover units that don’t represent typical ecosystems. In our study, urban areas, water bodies, and bare land harbored organisms including plants, but their numbers likely were comparably low and we therefore think that the effect of these areas was to an important degree abiotic. The identification and process-level investigation of such abiotic effects is beyond the scope of the present study. However, given that this is, to the best of our knowledge, a novel class of mechanisms that potentially underpin landscape-level diversity-functioning relationships, we think that it is useful to speculate about their nature and how they conceptually integrate into existing frameworks of mechanisms. Urban areas often show higher air temperatures, increased cloud formation and increased precipitation^50^. Although not an important source of primary productivity themselves, these entities interact with their surrounding and can affect plant productivity there through a range of mechanisms including advective heat transport^26^. Along similar lines, the scale shift from species to larger ecological entities such as ecosystems allows to implicitly account for anthropogenic effects such as the emission of volatile nitrogenous compounds from agriculture or urban areas, effects that have no proximal causal relationship to species numbers. Interestingly, some of these effects may be described in analogy to mechanisms that support biodiversity effects in plant communities. For example, beneficial climatic effects of neighboring land-cover units may be thought of as facilitation^51^. Further, land-cover units differ in qualities such as albedo or surface roughness, properties that can be considered as functional traits and which may be useful in predicting landscape-level functioning. Also, different land-cover types may respond asynchronously to environmental drivers, thereby temporally stabilizing landscape level functioning in a way comparable to what has been found in species-level field studies and modeling analyses of communities and meta-communities^52,53^. Overall, we therefore argue that diversity–functioning analyses at the local and landscape scale are driven by different though conceptually comparable mechanisms and provide complementary insights, and that at larger scale emergent, species-independent mechanisms may play an important role. A multi-scale perspective that allows for such emergent effects appears particularly important when considering today’s terrestrial land surface which is profoundly shaped by anthropogenic activities^54^.

Our analyses aimed at identifying empirical patterns of relationships between landscape richness and landscape functioning, but the question about the underlying mechanisms is both obvious and relevant. Because our focus was phenomenological and we did not study mechanisms in detail, we cannot evaluate these directly, but will discuss some possibilities and corresponding analyses. Specifically, we were interested whether (1) effects were driven by particular land-cover type combinations; (2) effects might be related to local within-ecosystem α-diversity of species, through causal links or indirect associations between species richness and landscape richness; and (3) whether the spatial configuration of land-cover units can explain variation in addition to the variation explained by landscape richness. In the following, we consider these possibilities separately.

Plot-scale BEF experiments have shown that species-richness effects can emerge from dominant effects of one or few particular species (or their combination), or from more even contributions by many species^1,36^. A prominent example for the first is the interaction of legumes with non-legume plant species which accounted for a large fraction of the productivity rise with species richness in several grassland experiments^17,18^. We therefore asked whether the presence of particular combinations of land-cover types also would explain important parts of the overall landscape richness–landscape functioning relationship that we observed. Because we could not break down the satellite-sensed landscape-level functions to contributions of single land-cover types within landscapes, we could not infer complementarity or selection effects using additive partitioning^36^. Instead, we used mechanistic diallel analysis^55^ (Supplementary Methods, Supplementary Discussion and Supplementary Fig. 1) to assess whether specific pairs of land-cover types systematically increased or decreased landscape functions. One parameter derived from this partitioning scheme, the so-called “specific combining ability” (SCA), assesses such interactive effects of pairs of land-cover types. The analysis of SCAs showed statistically significant positive effects of combining two different land-cover types, corroborating our finding that mixed landscapes on average performed better than single-land-cover type landscapes due to land-cover type complementarity. It further showed statistically significant effects of particular combinations of land-cover types on specific landscape functions, but these effects were inconsistent across blocks, landscape sizes, and landscape functions. Overall, interactions among the different land-cover types thus added up to a net positive contribution to productivity, but attributing these effects to specific land-cover combinations and mechanisms was not possible and will require further research. It is critical to understand that the higher productivity in mixed landscapes we report here is an average property and unlikely to apply to all land-cover combinations individually. These “overyielding” effects may rarely be transgressive^38^, in particular when functioning levels differ significantly between interacting land-cover types. For example, fragmenting a large continuous forest by urbanization will almost certainly reduce landscape productivity despite increased landscape richness (but the productivity of this intertwined landscape may exceed the productivity of a landscape in which the same total forest and urban areas are spatially separated).

In our study, we used landscape richness, i.e. the richness of land-cover types, to predict landscape functioning. However, the local species richness of ecosystems within these land-cover types certainly also varied and could have driven local BEF effects through mechanisms similar to the ones identified in experimental BEF studies. Understanding the correlation between landscape richness and local species richness therefore is important to understand the observed patterns in landscape functioning, which are the manifestation of compound effects at both scales. Because no species inventories were available for our study landscapes, we tentatively explored the general relationship between landscape richness and species richness using data from a national biodiversity monitoring initiative^56^ that reported, among other species-richness variables, the richness of forest plant species for 1 × 1 km plots and that of herbaceous plants for 10 × 10 m plots across Switzerland (Supplementary Methods, Supplementary Discussion and Supplementary Table 1). Landscape richness and species richness were only very weakly correlated (*r*^2^ = 0.008–0.018; Pearson correlation coefficients adjusted for blocks). This suggests that the landscape richness–landscape functioning effects we observed were unrelated to potential effects of species richness and most likely originated from larger scale interactions between land-cover units. Searching for more specific evidence of such species richness-independent effects (Supplementary Methods, Supplementary Discussion and Supplementary Fig. 2), we found that average growing-season productivity of forest-only landscapes decreased with the amount of land covered by water in their surroundings ($\overline {{\mathrm{EVI}}}$; *F*_1,367_ = 13, *P* < 0.001; 250 × 250 m plots). Conversely, the amount of agricultural land around these forests increased growing-season length (GSL; *F*_1,367_ = 10 and *F*_1,119_ = 22, *P* = 0.002 and *P* < 0.001, for 250 × 250 m and 500 × 500 m plots, respectively). These findings are compatible with the idea of spatial heat subsidies that are positive from agricultural land (due to low albedo, at least when ground cover is low) and negative early in the season for water surfaces due to their high heat capacity, but a conclusive identification would require more extensive and process-oriented investigations. Nevertheless, we think that these patterns highlight the importance to further investigate interactions between land-cover types^24,57^. These may represent a new class of mechanisms underpinning diversity effects at the landscape level and corresponding ecosystem services provided by diverse landscapes^5^.

In virtually all real-world landscapes, compositional diversity is intrinsically linked to configurational diversity. In other words, landscapes that differ in number of land-cover types also differ with respect to size, shape, and arrangement of these elements^58,59^. This suggests that some interactions among land-cover units and types may occur primarily along their interfaces, while others may depend more on unit size and arrangement. Unsurprisingly, landscape richness was highly correlated with measures of configurational diversity in our study (Supplementary Methods, Supplementary Discussion, Supplementary Table 2, Supplementary Figs. 3 and 4). Adding these as additional explanatory variables to our statistical models indicated that edge density (the total interface length between land-cover types per unit area) and the connectedness of land-cover units of the same land-cover type explained additional variance in landscape functions that was not explained by landscape richness alone.

Unlike randomized BEF experiments, the statistical analysis of observational BEF data is plagued by correlations among multiple potential drivers of functioning. Many of these originate from effects of environmental variables that vary at large spatial scales and influence both diversity and functioning at these scales. For example, in our study area temperature decreases strongly with altitude and is associated with lower primary productivity and shorter growing seasons; at the same time species richness decreases with altitude^35^. Such effects can be separated only to some extent using more sophisticated statistical models^35,60^. Here, we tried to avoid these problems by using a quasi-experimental approach that allowed land-cover type richness and land-cover type composition to be treated as design variables that were uncorrelated with each other and with major environmental variables^40^. Our study thus covers a middle ground between randomized experiments by retaining some of their favorable properties, and real-world systems by allowing for their inherent complexity that likely is important for their functioning^11^.

It has previously been established that within-ecosystem species diversity matters for ecosystem functioning and stability^1,2^. Our analysis suggests that a similar relationship between landscape diversity and landscape functioning exists. Although its mechanistic basis awaits future investigation, some of the effects we report here appear to depend at least in part on emergent mechanisms independent of species diversity. We contend that landscape-level diversity–functioning relationships deserve increased attention, not least because they underlie the delivery of ecosystem services to humans^5^.

## Methods

### Study design

**Fig. 5:**
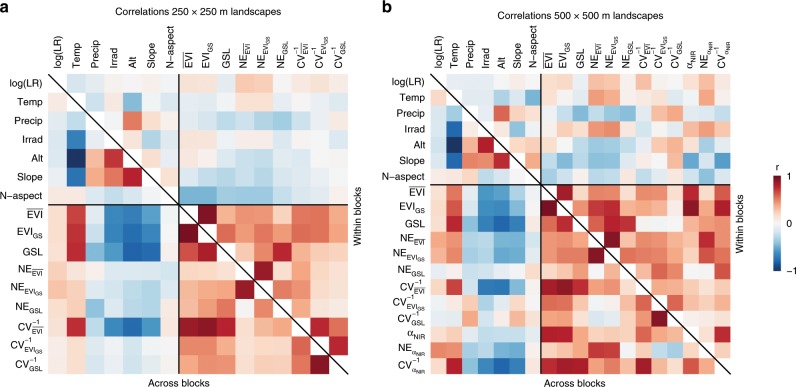
Correlations among landscape richness, environmental co-variables, and landscape functioning variables for 250 × 250 m and 500 × 500 m landscape plots. Matrices show overall (across block) and block-adjusted (within block) Pearson correlation coefficients (*r*) for 250 × 250 m landscapes (**a**) and 500 × 500 m landscapes (**b**). Adjusting for block reduces correlations of landscape richness and environmental variables (all n.s.). log(LR): log-transformed landscape richness; Temp: mean annual temperature; Precip: annual precipitation; Irrad: mean irradiation; Alt: altitude; Slope: slope; N-aspect: north-south aspect; $\overline {{\mathrm{EVI}}}$ and EVI_GS_: mean and integrated growing-season productivity; GSL: growing-season length; α_NIR_: near-infrared albedo; NE: net diversity effect for these variables; CV^−1^: temporal stability of these variables; *n* = 237 and 77 for 250 × 250 m and 500 × 500 m land-cover composition × block combinations, respectively. Source data are provided as a Source Data file.

We established two networks of plots that contained landscapes with a spatial extent of either 250 × 250 m or 500 × 500 m; their boundaries were congruent with MODIS 250 m and 500 m Vegetation Index pixels^31^, respectively. The plot networks covered the entire area of Switzerland (41,248 km^2^) and spanned an altitudinal range of 193 to 3279 m above sea level. To account for regional variation in environmental conditions, we divided the study area into six biogeographic regions (BGR) that form distinct units with respect to climate, edaphic conditions, and distribution patterns of fauna and flora^34^. We then subdivided the six BGRs by altitude, using 500 m increments (Fig. 1a). Not all land-cover types and type combinations occurred in the 36 established BGR × altitude blocks (Fig. 1b). We therefore used a nested design with independent gradients of land-cover type richness, hereafter referred to as landscape richness (LR), in each of these BGR × altitude blocks. Within each block, we determined the largest set of land-cover types that still allowed spanning a wide gradient in landscape richness with all possible land-cover combinations (i.e. land-cover compositions) realized. Hence, landscape richness gradient and average land-cover abundance were orthogonal (Fig. 1b). Using an optimization procedure, we attempted to select 24 replicates for each land-cover composition in each block. This procedure further (1) maximized the minimum pairwise distance among all selected landscapes within a block, ensured (2) that landscapes of identical land-cover composition were at least 1 km apart, (3) that the mean altitude, slope, and north-south aspect were as equally distributed as possible in all land-cover compositions, and (4) that these values and the fractional cover of land-cover types showed as little correlation as possible with landscape richness. If not enough landscapes could be found that satisfied these criteria, we lowered the number of replicates per land-cover composition in this particular block and landscape richness level. Only plots with land-cover fractions that deviated by less than 50% (250 × 250 m landscape plots) or 80% (500 × 500 m landscape plots) from perfect evenness were considered for the optimization. The final data sets encompassed 4186 (250 × 250 m) and 788 landscape plots (500 × 500 m), representing 35 and 27 unique land-cover compositions, respectively, (237 and 77 unique land-cover composition × block combinations; Fig. 1b). Importantly, these sets of plots showed no significant correlation of landscape richness with the fractional cover of any land-cover type within or across blocks (Supplementary Fig. 3), i.e. land-cover type richness and the abundance of particular land-cover types were orthogonal for any practical purpose. Blocking also minimized any correlation of landscape richness with environmental factors that could co-drive productivity or other landscape functions (Fig. 5).

### Landscape richness (LR)

Landscape richness (LR) was determined as the number of land-cover types found in a landscape. Land cover information was taken from 100-m spatial resolution point data available from the Swiss Federal Statistical Office (GEOSTAT, product name: NOAS04). The originally 17 land-cover types were aggregated into the 8 land-cover types forest (original classes: forest [except brush]; brush forest; woods), grassland (meadows, farm pastures, alpine meadows, alpine pastures), arable (vineyard and horticulture; orchard; arable), urban (industrial and commercial; building; transportation; special urban), urban green (recreational and cemeteries), water (lakes; rivers), unproductive (unproductive vegetation), and bare land (bare land; glaciers, perpetual snow). We determined the fractional cover of each land-cover type by clipping land-cover pixels at the boundaries of the study landscapes defined by the MODIS Vegetation Index pixels.

### Primary productivity and phenology

We used space-borne MODIS Vegetation Index data (MOD13Q1 and MOD13A1^31^) with 250 m and 500 m spatial resolution and 16-day temporal resolution to derive land-surface phenology and growing-season productivity metrics. Specifically, we used the EVI which quantifies photosynthetically active vegetation from the ratio of reflected red and near-infrared light; it is similar to the normalized difference vegetation index (NDVI) but more robust because it uses blue reflectance data to reduce bias from aerosols^61^. We derived the vegetation growing season for every study landscape and year (2000–2016) by smoothing EVI time series for the respective year using a modified version of the harmonic analysis of time series algorithm (HANTS; refs. ^35,62^). More specifically, we fitted a model consisting of three dominant harmonics and an intercept to the observed EVI time series [EVI(*ω)* ~ *α*_1_cos(*ω* + *φ*_1_) + *α*_2_cos(2*ω* + *φ*_2_) + *α*_3_cos(4*ω* + *φ*_3_) + *β*]. As specified by the HANTS algorithm, observed data with residuals exceeding specific thresholds (0.5, 0.2, 0.1 and 0.05 raw EVI values) were replaced by model predictions and the procedure repeated. Growing-season length (GSL) was determined using the NDVI-ratio method^63^, which defines the growing season as the time of the year when vegetation activity exceeds the mean of its annual minimum and maximum value (ref. ^35^). GSL is a commonly used land surface phenology metric for large spatial scales^64,65^. We used two primary productivity metrics; the first, $\overline {{\mathrm{EVI}}}$, equals the average growing-season EVI. The second integrates EVI over the growing season: EVI_GS_ = $\mathop {\smallint }\nolimits_{{\mathrm{SOS}}}^{{\mathrm{EOS}}} {\mathrm{EVI}}\left( t \right){\mathrm{d}}t = \overline {{\mathrm{EVI}}} \cdot {\mathrm{GSL}}$. We further determined growing-season peak EVI (EVI_max_). EVI values are dimensionless, and so are $\overline {{\mathrm{EVI}}}$ and EVI_max_; EVI_GS_ and GSL are shown in units of years (yr) and days (d), respectively.

### Land-surface albedo

We used space-borne land surface albedo (α; dimensionless) data with a spatial resolution of 500 m (MODIS MCD43A3^32^). These data are calculated from multi-date (16 days), multi-band, cloud-free, atmospherically-corrected surface reflectance. We focused on white-sky albedo (WSA) because it is an inherent property of the land surface and is used as input to climate models^66^. Specifically, we used the 17-year average of mean summer (June to August) shortwave WSA (α_SW;_ 0.3–5.0 µm wavelength) and its two components visible WSA (α_VIS_; 0.3–0.7 µm) and near-infrared WSA (α_NIR;_ 0.7–5.0 µm). The partitioning of α_SW_ into α_VIS_ and α_NIR_ is necessary because of the marked difference of vegetation reflectance in these spectral domains^67^.

### Stability

We quantified the temporal stability of all productivity and albedo metrics as inverse of the coefficient of variation (CV^−1^), i.e. as mean of the annual values divided by the standard deviation of the annual values (2000–2016; *n* = 17). This is a commonly used measure of population, community or ecosystem stability in ecological studies^1,13,68,69^. Although other metrics of stability exist^70,71^ we used CV^−1^ (1) because it allows direct comparison with other BEF studies, and (2) because in our case variation in functioning likely was the result of a range of environmental factors, many of which remain unknown, precluding the use of metrics that depend on timing and magnitude of a specific disturbance.

### Net diversity effects (NE)

NE values were calculated as the observed value of a metric in a mixed landscape (landscape richness > 1) minus its expected value^36^. The expected value was the weighted mean of the average values of the corresponding single land-cover type landscapes (landscape richness = 1) in the respective block; as weights we used the proportion of land covers in the mixed landscape. NE was determined within blocks. NE quantifies the change in a metric with an increase in landscape richness, while adjusting for land-cover composition. For productivity terms, NE often also is referred to as “overyielding”^37,38^.

### Environmental co-variables

The topographic co-variables altitude (Alt; m above sea level), slope (Slope; degrees inclination) and north-south-component of the slope (N-aspect; positive and negative values for north- and south-facing slopes, respectively) were calculated from a digital elevation model with 25 m spatial resolution (product DHM25; Swiss Federal Office of Topography swisstopo) and averaged per landscape plot. Climatic co-variables were mean annual temperature (Temp; in °C, average of years 2000–2014), annual precipitation (Precip; in mm, average of years 2000–2014), and mean annual surface incoming short-wave radiation (Irrad; in W m^−2^, average of years 2004–2012). These data were calculated from interpolated gridded monthly data sets [0.0208° spatial resolution (~2.3 km in east-west and 1.6 km in north-south direction), Swiss Federal Office of Meteorology and Climatology MeteoSwiss, products TabsM, RhiresM, and msg.SIS.M].

### Statistical analysis

We tested effects of landscape richness using general linear mixed models summarized by analysis of variance (ANOVA; ASReml-R package^72^; VSN International, Hemel Hemsted, UK; R 3.5; http://r-project.org). Fixed effects were, in this order, BGR, ALT, BGR × ALT (BGR: biogeographic regions; ALT: altitude interval; the terms so far specify the blocks), log-transformed landscape richness [log(LR)], and the fraction of low-productivity land-cover types (fracL; see below). Land-cover composition was a random term defining the replication level for landscape richness. We evaluated different transformations of landscape richness and found that the natural logarithm of landscape richness [log(LR)] fitted the data slightly better than untransformed landscape richness, although differences in Akaike’s information criterion were small (ΔAIC = 1.3, average across all response variables). Models with quadratic and cubic transformations of landscape richness performed substantially worse (ΔAIC = 4.7 and 7.5, respectively). Land-cover types fell into two main groups that differed systematically in productivity. To adjust model residuals for this systematic, non-random, effect, we included the land-cover fraction of low-productivity land-cover types (fracL) as additional term after log(LR); low-productivity land-cover types were water, bare land, urban and unproductive vegetation. For the analysis of net diversity effects (NE), we first tested whether mixed landscapes performed better than single land-cover type landscapes (NE > 0; one-sample t-test), followed by a test whether NE increased with log(LR) by using linear mixed models as described above but excluding fracL because the correction for low-productivity land-cover types is inherent in the calculation of NE.

Given the different land-cover compositions in 250 × 250 m and 500 × 500 m landscape plots, we analyzed these data sets separately, except when testing for the scale-dependence of effects where we included the fixed effects terms SCALE and log(LR) × SCALE plus the additional random-effects term land-cover composition × SCALE. Prior to analysis, we aggregated all dependent variables to a single value for each land-cover composition × block combination, which reduced data from the original number of study units to 237 (35 unique land-cover compositions) for 250 × 250 m landscape plots and to 77 (27 unique land-cover compositions) for 500 × 500 m landscape plots.

For all effects of log(LR), we derived normalized effect sizes (*Z*_r_ = $\frac{1}{2}{\mathrm{log}}_e\left( {\frac{{1 + r}}{{1 - r}}} \right)$ with *r* *=* $\sqrt {\frac{F}{{F + {\rm{d}}f}}}$; *F* = ANOVA-table F-value with df denominator degrees of freedom; ref. ^39^) and their corresponding asymptotic variance (*V*_z_ $= \frac{1}{{n - 3}}$, *n* = total sample size^39^). *Z*_r_ values allow comparison with virtually any study because they can be derived from many summary statistics. To compare these meta-analytical effect sizes to the ones from plot-scale BEF experiments^4^, we calculated average *Z*_r_ values for plant species-richness effects on aboveground biomass production (*n* = 13 experiments^4,73^) and its temporal stability (*n* = 3 experiments^13,68,69^) in a range of grassland biodiversity field experiments.

### Reporting summary

Further information on research design is available in the Nature Research Reporting Summary linked to this article.

## Supplementary information

Supplementary Information

Peer Review File

Reporting Summary

## Data Availability

The data used in this study are available from Dryad (10.5061/dryad.gb5mkkwkj). Data used to produce Figs. 2–5 are provided as a Source Data file.

## Source data

Source Data

## Code availability

The computer code used to run the analyses is provided as a Source Data file.
