# Supplementary material for: Terrestrial land-cover type richness is positively linked to landscape-level functioning
Source: Nat Commun. 2020 Jan 9;11:154. doi: 10.1038/s41467-019-14002-7 (PMC6952349; doi:10.1038/s41467-019-14002-7)
Supplement: Supplementary file 1 — Supplementary Information [file 41467_2019_14002_MOESM1_ESM.pdf]

Supplementary Information

# Terrestrial land-cover type richness is positively linked to landscape-level functioning

Oehri et al.

**Correspondence:**
Jacqueline Oehri and Pascal A. Niklaus
Department of Evolutionary Biology and Environmental Studies, University of Zurich, Winterthurerstrasse 190, 8057 Zurich
ph. +41 44 635 4402, jacqueline.oehri@ieu.uzh.ch
ph. +41 44 635 3413, pascal.niklaus@ieu.uzh.ch

# Contents

**Supplementary Methods** 3

Pairwise interactions among land-cover units 3

Correlation of landscape richness with species richness 4

Long-range interactions among land-cover types 6

Spatial configurational diversity of land-cover units 6

**Supplementary Discussion** 8

Pairwise interactions among land-cover units 8

Correlation of landscape richness with species richness 9

Long-range interactions among land-cover types 9

Spatial configurational diversity of land-cover units 9

**Supplementary Tables** 11

Supplementary Table 1 11

Supplementary Table 2 12

**Supplementary Figures** 13

Supplementary Figure 1 13

Supplementary Figure 2 14

Supplementary Figure 3 15

Supplementary Figure 4 16

**Supplementary References** 17

# Supplementary Methods

## Pairwise interactions among land-cover units

We analyzed pairwise interactions of land-cover units by mechanistic diallel analysis[1]. Diallel analysis was originally developed to model the performance of crosses of parental plant accessions as a function of i) the general contributions of the parents (GCA; general combining ability), and ii) contributions explained by the specific combination of the parental lines (SCA; specific combining ability). This method can be generalized to investigate any orthogonal setting of units composed of one or two components, where units with one component correspond to the case where both parental lines are identical. In the diallel model $y_{a,b,i} \sim GCA_a + GCA_b + SCA_{a,b} + \varepsilon_{a,b,i}$ where $y_{a,b,i}$ is the performance of the unit comprising component a and b in replicate *i*, $GCA_n$ is the average contribution of the component n to the general performance of units across the study, $SCA_{a,b}$ is the average contribution of the specific combination of components a and b, and $\varepsilon_{a,b,i}$ is the corresponding residual. In this model, $GCA_a + GCA_b$ is the expected performance of the combination of a and b (with a=b when two identical units are combined), and $SCA_{a,b}$ measures the deviation from this expectation, i.e. the net interaction of a and b. The diallel framework thus allows to identify positive or negative interactions between two components of a unit, similar to the additive partitioning method of Loreau & Hector[2]. An advantage of the diallel method is that it can be applied to cases where data is available at the whole unit level but not separately for the individual components a and b. A further difference to the additive partitioning is that SCAs are corrected for differences in the average performance of each unit across combinations, which is not the case for the NE and CE in the additive partitioning method.

We determined GCA and SCA values by fitting a mechanistic diallel model to the subset of our data where landscape richness ≤ 2, separately for every block. Conceptually, single land-cover landscape plots were treated as combination of twice the same land-cover type. The model matrix was constructed by superimposing effects of the two component land-cover types (i.e. both GCA terms) using the and() special function of ASReml (VSN International, Hemel Hemsted, UK; see ref.[3]).

For the subsequent analyses, we used these block-wise GCA and SCA values as new dependent variables. We tested whether SCAs differed between single land-cover landscapes and mixed landscapes using a model with landscape richness as fixed effect. We then tested whether GCAs depended on land-cover type. Similarly, we tested whether SCAs depended on land-cover pairs and, if significant, performed pair-wise multiple comparisons tests (Tukey's HSD, function glht in R-library multcomp[4]).

**Correlation of landscape richness with species richness**

We tested for correlations between local species richness and land-cover type richness (i.e. landscape richness) in our study area using data from the Swiss Biodiversity Monitoring Program (BDM; biodiversitymonitoring.ch; refs.[5,6]). The presence of vascular plant species within ecosystems ("Z9 Indicator"; ref.[6]) and within landscapes ("Z7 Indicator"; ref.[6]) was recorded in five–year intervals since the year 2001. The Z9 data were recorded in over 1400 circular 10m$^2$ plots. The Z7 data was recorded along two transects across more than 500 landscape plots of 1 km$^2$ in size. Z7 and Z9 plots both are regularly spread across Switzerland.

We calculated herbaceous α-species richness in the Z9 plots classified as meadow or another form of grassland ($S_{grass}$) for the 2001–2016 period. The 10-m$^2$ Z9 plots were too small to reasonably assess the species richness of woody species, including trees, and we therefore used woody species richness along the Z7 transects ($S_{forest}$). In principle, such a transect could cross different land-cover types, but woody species occur primarily in forest and the land-cover type forest varies mostly at larger environmental and spatial scales. We therefore consider these data a reasonable approximation of α-richness of woody forest species.

We then determined landscape richness for the Z7 and Z9 plots. For the 10-m$^2$ Z9 plots, we calculated landscape richness in quadratic areas 250×250 m and 500×500 m in size with the Z9 plot in their center. For the 1 km$^2$ Z7 plots, we calculated landscape richness for a 250×250 m (and 500×500 m) area basis by dividing the Z7 plots into 16 (and 4) quadrats and averaging the landscape richness values obtained for these sub-quadrats.

Next, we removed all Z9 plots and Z7 sub-quadrats that we would not have selected in our main study design because (1) the evenness of land-cover types did not comply with the selection criterion used in the main study, (2) the plot contained compositions that were not included in the main data set (e.g. the land-cover type “urban green”), (3) plots had landscape richness >4, (4) plots had altitude, slope inclination, or north aspect of the slope values outside the range used in the main analysis, or (5) plots were in blocks that were not part of the main analysis (see Figure 1b in main text). Partial correlation coefficients between landscape richness and Z9 or Z7 species richness were calculated after adjusting for block effects (Supplementary Table 1).

**Long-range interactions among land-cover types**

Functioning metrics were only available at the level of entire landscape plots. Complementing the diallel analysis, we tested for effects of surrounding land-cover types on landscape plots containing a single land-cover type (landscape richness=1). Forests were the only such landscapes that were dominated by vegetation and occurred in enough replicates (n=390 and 131 for 250×250 m and 500×500 m landscape plots, respectively). We characterized the surrounding of these forest landscapes by the fractional amount of each of the eight aggregated land-cover types (see Methods in the main text). For 250×250 m landscape plots, we focused on a circular area with radius 500 m from the center of the landscape plot. For 500×500 m landscape plots, this radius was increased to 1000 m. We tested for effects of the fractional cover of each surrounding land-cover type using a linear model with block followed by the fractional cover of the land-cover type under consideration.

**Spatial configurational diversity of land-cover units**

We determined metrics characterizing the spatial configurational diversity of land-cover units (Supplementary Table 2) within all 250×250 m and 500×500 m landscape plots. These were edge density (ED), effective mesh size (MESH), Simpson's diversity index (SIDI) and patch cohesion index (COHESION).

Next, we determined unadjusted and block-adjusted partial Pearson correlations between these variables, including our design variable log(LR) (i.e. log-transformed landscape richness, see Methods, main text) and the landscape functioning variables

productivity, albedo and their temporal stability. We then tested the effects of configurational diversity on the landscape functioning variables by using these metrics instead of log(LR) (models described in Methods, main text). Finally, we tested whether configurational diversity metrics could explain variation in landscape functioning variables if fitted after log(LR) in the statistical models, i.e. whether configurational diversity could explain additional variation not already explained by log(LR).

## Supplementary Discussion

### Pairwise interactions among land-cover units

Neither GCAs nor SCAs differed between 250×250 m and 500×500 m landscape plots (GCA: $F_{1,119}<1$, n.s.; SCA: $F_{1,124}<2.8$; n.s.). However, GCAs differed among land-cover types ($F_{6,30}\geq 12.0$, $P<0.001$, for $\alpha_{NIR}$ and $CV^{-1}_{\alpha_{NIR}}$; $F_{6,114}\geq 6.4$, $P<0.001$, for all other landscape functions; Supplementary Figure 1a,b). This indicates that land-cover types contributed differently to landscape functioning.

Except for $CV^{-1}_{EVI_{GS}}$, SCA values also differed among land-cover pairs ($F_{11,18}\geq 3.5$, $P<0.01$ for $\alpha_{NIR}$ and $CV^{-1}_{\alpha_{NIR}}$; $F_{14,111}\geq 2.0$, $P<0.05$ for all other landscape functions). The presence of the urban land-cover type in the pair resulted in significantly positive SCA values ($F_{1,94}=12.9$; $P<0.001$; Supplementary Figure 1c) in the case of $\overline{EVI}$ in 250×250 m landscape plots. Similarly, the presence of water increased SCA values for temporal stability of productivity ($CV^{-1}_{\overline{EVI}}$; $F_{1,95}=10.1$; $P<0.01$; Supplementary Figure 1d). However, in general, the differences among SCAs of land-cover pairs changed with block (Tukey tests). Patterns in SCA also varied among landscape functions (Supplementary Figure 1c,d).

In accordance with our analysis of the main text, SCAs were significantly higher in mixed compared to single land-cover type landscape plots for the primary productivity variables and their temporal stability ($\overline{EVI}$, $EVI_{GS}$,$CV^{-1}_{\overline{EVI}}$, and $CV^{-1}_{EVI_{GS}}$) for the 250×250 m landscapes (all $F_{1,19}\geq 5.8$; $P\leq 0.026$). These effects were similar but less significant in the 500×500 m landscapes.

### Correlation of landscape richness with species richness

For both 250×250 m and 500×500 m landscapes we found that Person correlation coefficients between landscape richness and species richness of grassland and forest plants ($S_{grass}$ and $S_{forest}$, respectively) were relatively small (Supplementary Table 1).

### Long-range interactions among land-cover types

Testing the effects of surrounding land-cover types on landscape functioning of forest landscapes (Supplementary Figure 2a), we found that in 250×250 m landscape plots, the fractional cover of water ($W_{frac}$) surrounding forest landscape plots was negatively related to average growing-season productivity ($\overline{\mathrm{EVI}}$; $F_{1,367}$=13, P<0.001; Supplementary Figure 2b). Conversely, the surrounding fractional cover of agricultural area ($A_{frac}$, within 500 m of the center of forest landscape plots) was positively related to growing-season length (GSL) in these same landscapes ($F_{1,367}$=10, P=0.002; Supplementary Figure 2c). In 500×500 m forest landscape plots, GSL increased with $A_{frac}$ ($F_{1,119}$=22, P<0.001; Supplementary Figure 2c).

### Spatial configurational diversity of land-cover units

The different configurational diversity metrics were highly correlated across and within blocks. Edge density (ED) and the Simpson's diversity index (SIDI) increased with landscape richness [log(LR)], while effective mesh size (MESH) and patch cohesion index (COHESION) decreased with log(LR) (Supplementary Figure 3). All metrics explained similar amounts of variance in landscape functioning variables (Supplementary Figure 4).

However, only edge density and patch cohesion index explained significant additional amounts of variation for some of the dependent variables when fitted after log(LR) (edge density: for $\mathrm{CV}_{\overline{\mathrm{EVI}}}^{-1}$, $\mathrm{CV}_{\mathrm{EVI_{GS}}}^{-1}$ in 250×250 m landscapes and $\alpha_{NIR}$ in 500×500 m landscapes; patch cohesion index: for $\mathrm{NE}_{\mathrm{EVI_{GS}}}$ and $\mathrm{CV}_{\mathrm{EVI_{GS}}}^{-1}$ in 500×500 m landscapes). As a result of the plot selection procedure in our quasi-experimental design, none of the configurational diversity metrics was significantly correlated with the fractional cover of any land-cover type (Supplementary Figure 3).

# Supplementary Tables

## Supplementary Table 1

**Supplementary Table 1. Correlation between landscape and plant species richness.** Grassland species richness ($S_{grass}$) refers to herbaceous species found in 10 $m^2$ plots. Woody species richness ($S_{forest}$) refers to woody species found along transects across 1 $km^2$ plots. See Supplementary Methods for details. n: number of study units; r: correlation coefficients, $r_{partial}$: partial correlation coefficients. r and $r_{partial}$ were calculated after adjusting for block effects.

| Species richness | Landscape plot size | n | $r^2$ | $r^2_{partial}$ |
|---|---|---|---|---|
| $S_{grass}$ | 250 × 250 m | 238 | <0.001 | 0.012 |
| | 500 × 500 m | 120 | 0.008 | 0.009 |
| $S_{forest}$ | 250 × 250 m | 382 | 0.018 | 0.021 |
| | 500 × 500 m | 240 | 0.015 | 0.011 |

## Supplementary Table 2

**Supplementary Table 2. Landscape configurational diversity metrics.** All definitions are taken from McGarigal[7].

| Landscape configuration measures | Description |
|---|---|
| Edge density (ED, $m^{-1}$) | Total length of edges (interface between land-cover units) divided by the total landscape area. |
| Effective mesh size (MESH, $m^2$) | The sum of the squared patch areas divided by the plot area. MESH increases with average land-cover unit size. |
| Simpson's diversity index (SIDI) | Simpson's diversity index, calculated using the area of each land-cover as measure of abundance. |
| Patch cohesion index (COHESION) | COHESION quantifies the connectedness within a land-cover type. According to ref.[5], this index was designed "to quantify the connectivity of habitat as perceived organisms dispersing in a (…) landscape". Here, we use the average COHESION index of the land-cover types present. |

# Supplementary Figures

## Supplementary Figure 1

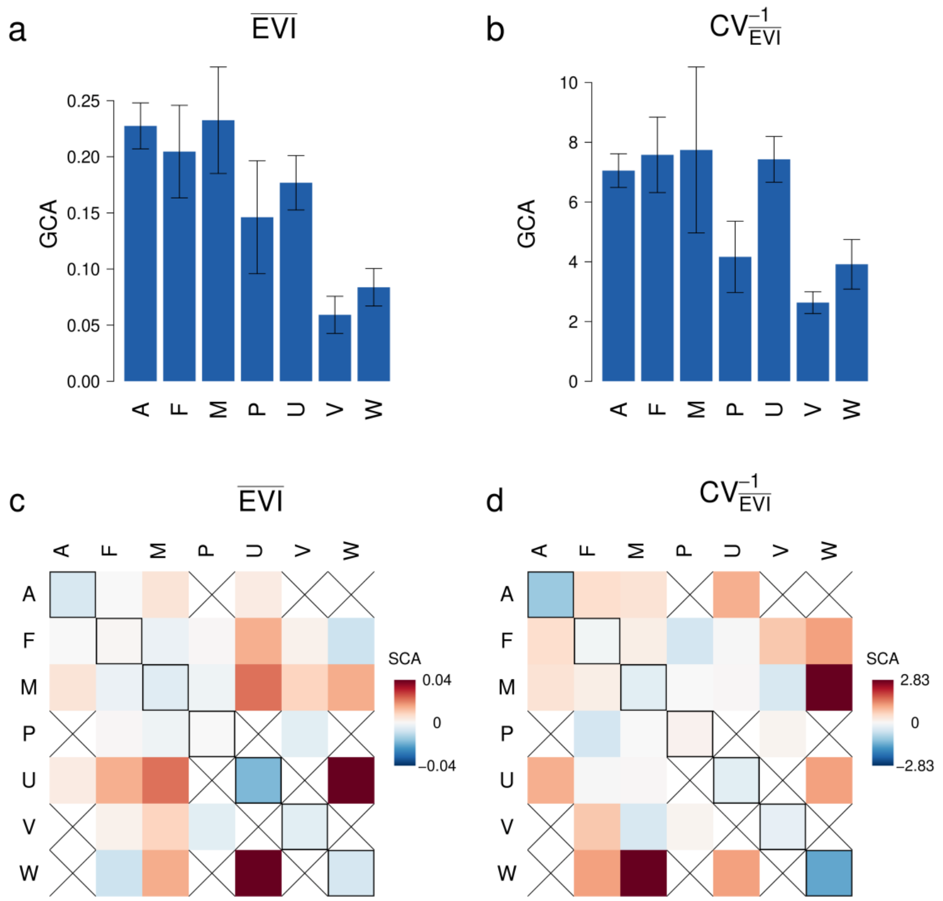


**Supplementary Figure 1 | General (GCA) and specific (SCA) combining abilities in 250×250 m landscape plots.** GCA values for a primary productivity proxy [$\overline{\mathrm{EVI}}$; **(a)**], and its temporal stability [$\mathrm{CV}_{\overline{\mathrm{EVI}}}^{-1}$; **(b)**; means across blocks ± s.d.] differ depending on land-cover type (n=84). SCA values for a primary productivity proxy [$\overline{\mathrm{EVI}}$; **(c)**], and its temporal stability [$\mathrm{CV}_{\overline{\mathrm{EVI}}}^{-1}$; **(d)**; means across blocks] for all land-cover pairs (n=180). A: arable, F: forest, M: grass, P: unproductive, U: urban, V: bare land, W: water. Crosses indicate that land-cover type combination is absent from all blocks.

**Supplementary Figure 2**

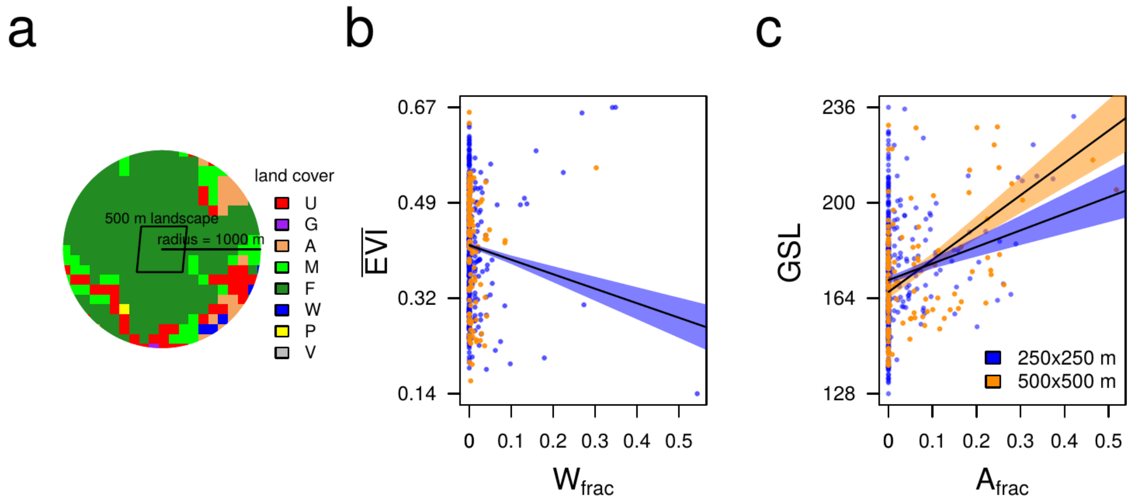


**Supplementary Figure 2 | Effects of different land-cover type fractions in the surrounding of forest landscapes. (a)** Example of a 500×500 m forest landscape plot with its 1,000-m surrounding radius. **(b)** Dependency of forest productivity (approximated with $\overline{\mathrm{EVI}}$) on the fraction of water ($W_{frac}$) in the surrounding. **(c)** Dependency of vegetation growing season length (GSL) on the fractional cover of agricultural land ($A_{frac}$) in the surrounding [radius of 500 and 1000 m in 250×250 m (n=390) and 500×500 m (n=131) landscape plots, respectively]. Black line and shaded areas are model-predicted mean ± s.e.m.; A: arable, F: forest, M: grass, P: unproductive, U: urban, V: bare land, W: water.

## Supplementary Figure 3

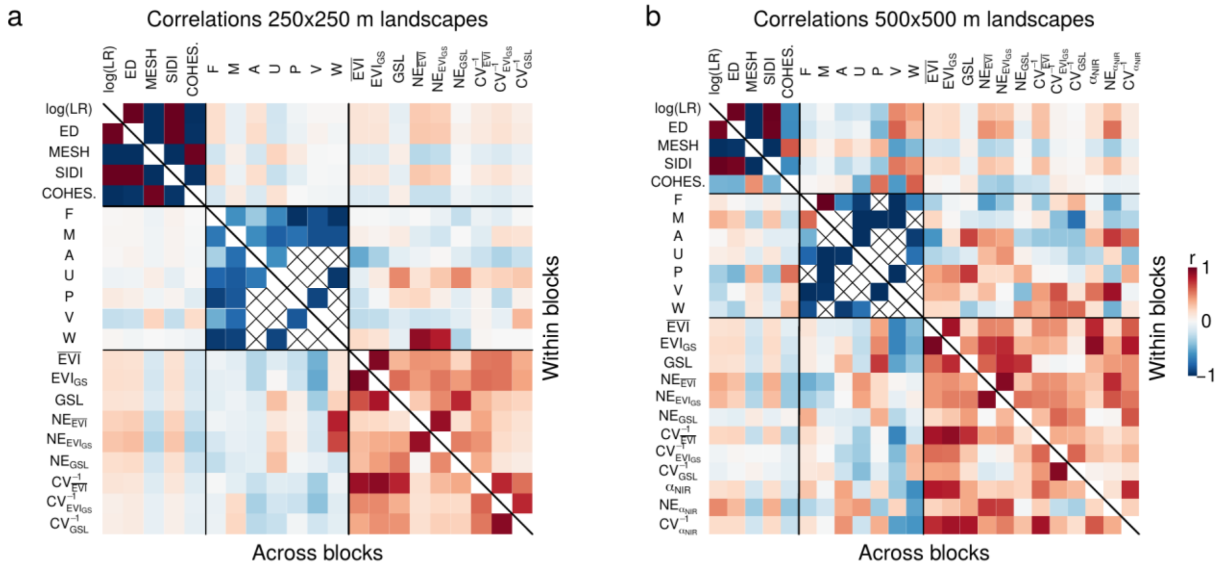


**Supplementary Figure 3 | Correlations between landscape richness, landscape configurational diversity, relative fraction of land-cover types, and landscape functioning.** Correlation matrices show overall (across block) and block-adjusted (within block) Pearson correlation coefficients (r) for 250×250 m landscapes **(a)** and 500×500 m landscapes **(b)**. log(LR): log-transformed landscape richness; for abbreviations of configurational diversity variables (i.e. ED, MESH, SIDI, COHES.) see Supplementary Table 2. Abbreviations for relative fractions of land-cover types: A = arable, F = forest, M = grass, P = unproductive, U = urban, V = bare land, W = water. Landscape functioning variables: $\overline{\text{EVI}}$ and $\text{EVI}_{GS}$ = mean growing-season and integrated growing-season productivity, GSL = growing-season length, $\alpha_{NIR}$= near-infrared albedo, NE = net diversity effect of corresponding landscape functioning variable, $CV^{-1}$ = temporal stability of corresponding landscape functioning variable. Crosses indicate that land-cover type combination is absent from all blocks. n=237 and 77 land-cover composition x block combinations for 250×250 m and 500×500 m landscape plots, respectively.

**Supplementary Figure 4**

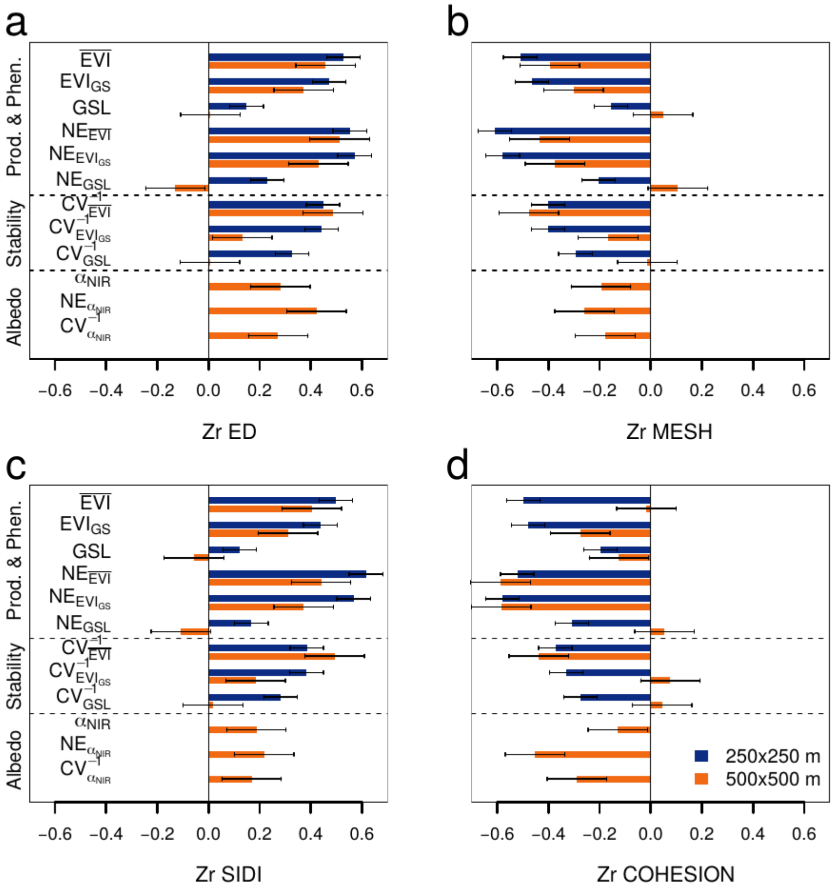


**Supplementary Figure 4 | Normalized effect sizes ($Z_r$) of configurational diversity metrics for all landscape functioning variables.** *Z*r values are shown for ED: edge density **(a)**, MESH: effective mesh size **(b)**, SIDI: Simpson's diversity Index **(c)**, and COHESION: patch cohesion index **(d)**. Landscape functioning variables: $\overline{\mathrm{EVI}}$ and $EVI_{GS}$ = mean growing-season and integrated growing-season productivity, GSL = growing-season length, $\alpha_{NIR}$= near-infrared albedo, NE = net diversity effect of corresponding landscape functioning variable, $CV^{-1}$ = temporal stability of corresponding landscape functioning variable. Bars show mean ± s.d. of *Zr* values. n=237 and 77 for 250×250 m (blue) and 500×500 m (orange) landscape plots, respectively. See Supplementary Table 2 for a detailed explanation of configurational diversity metrics.

## Supplementary References


1 Griffing, B. Concept of general and specific combining ability in relation to diallel crossing systems. Aust. J. *Biol. Sci.* **9**, 463–493, doi: 10.1071/BI9560463 (1956).

2 Loreau, M. & Hector, A. Partitioning selection and complementarity in biodiversity experiments. *Nature* **412**, 72-76, doi:10.1038/35083573 (2001).

3 Butler, D. asreml: asreml() fits the linear mixed model. *R package version 3.0*. www.vsni.co.uk (2009).

4 Hothorn, T., Bretz, F. & Westfall, P. Simultaneous Inference in General Parametric Models. *Biom. J.* **50**, 346-363, doi: 10.1002/bimj.200810425 (2008).

5 Weber, D., Hintermann, U. & Zangger, A. Scale and trends in species richness: considerations for monitoring biological diversity for political purposes. *Glob. Ecol. Biogeogr.* **13**, 97-104, doi:10.1111/j.1466-882X.2004.00078.x (2004).

6 BDM Coordination Office (2014) Swiss Biodiversity Monitoring BDM. Description of Methods and Indicators, 1–104 (Federal Office for the Environment, Bern, Switzerland, 2014).

7 McGarigal K (2015) Fragstats Help. Version 4.2. Available at www.umass.edu/landeco/research/fragstats/documents/fragstats.help.4.2.pdf. Accessed August 17, 2017 (University of Massachusetts, Amherst, MA, 2015).
