# Supplementary material for: Terrestrial land-cover type richness is positively linked to landscape-level functioning
Source: Nat Commun. 2020 Jan 9;11:154. doi: 10.1038/s41467-019-14002-7 (PMC6952349; doi:10.1038/s41467-019-14002-7)
Supplement: Supplementary file 3 — Reporting Summary [file 41467_2019_14002_MOESM3_ESM.pdf]

nature research

Corresponding author(s): Pascal A. Niklaus & Jacqueline Oehri

Last updated by author(s): Nov 18, 2019

Reporting Summary

Nature Research wishes to improve the reproducibility of the work that we publish. This form provides structure for consistency and transparency in reporting. For further information on Nature Research policies, see Authors & Referees and the Editorial Policy Checklist .

Statistics

For all statistical analyses, confirm that the following items are present in the figure legend, table legend, main text, or Methods section.

n/a	Confirmed	
☐	☒	The exact sample size (*n*) for each experimental group/condition, given as a discrete number and unit of measurement
☐	☒	A statement on whether measurements were taken from distinct samples or whether the same sample was measured repeatedly
☐	☒	The statistical test(s) used AND whether they are one- or two-sided<br>*Only common tests should be described solely by name; describe more complex techniques in the Methods section.*
☐	☒	A description of all covariates tested
☐	☒	A description of any assumptions or corrections, such as tests of normality and adjustment for multiple comparisons
☐	☒	A full description of the statistical parameters including central tendency (e.g. means) or other basic estimates (e.g. regression coefficient) AND variation (e.g. standard deviation) or associated estimates of uncertainty (e.g. confidence intervals)
☐	☒	For null hypothesis testing, the test statistic (e.g. *F*, *t*, *r*) with confidence intervals, effect sizes, degrees of freedom and *P* value noted<br>*Give P values as exact values whenever suitable.*
☒	☐	For Bayesian analysis, information on the choice of priors and Markov chain Monte Carlo settings
☐	☒	For hierarchical and complex designs, identification of the appropriate level for tests and full reporting of outcomes
☐	☒	Estimates of effect sizes (e.g. Cohen's *d*, Pearson's *r*), indicating how they were calculated

Our web collection on statistics for biologists contains articles on many of the points above.

Software and code

Policy information about availability of computer code

Data collection	All data were collected using custom code in R (version 3.5; http://r-project.org) and several open-source libraries described where appropriate in the main manuscript text
Data analysis	All data were analyzed using the ASReml-R package (VSN International, Hemel Hemsted, UK) and R (version 3.5; http://r-project.org) as described in the main manuscript text

For manuscripts utilizing custom algorithms or software that are central to the research but not yet described in published literature, software must be made available to editors/reviewers. We strongly encourage code deposition in a community repository (e.g. GitHub). See the Nature Research guidelines for submitting code & software for further information.

Data

Policy information about availability of data

All manuscripts must include a data availability statement. This statement should provide the following information, where applicable:

- Accession codes, unique identifiers, or web links for publicly available datasets
- A list of figures that have associated raw data
- A description of any restrictions on data availability

The data used in this study are available from Dryad (DOI: https://doi.org/10.5061/dryad.gb5mkkwkj). The data used to produce Figures 2—5 are provided as a Source Data file.

Field-specific reporting

Please select the one below that is the best fit for your research. If you are not sure, read the appropriate sections before making your selection.

☐ Life sciences ☐ Behavioural & social sciences ☒ Ecological, evolutionary & environmental sciences

For a reference copy of the document with all sections, see nature.com/documents/nr-reporting-summary-flat.pdf

Ecological, evolutionary & environmental sciences study design

All studies must disclose on these points even when the disclosure is negative.

Study description	We assessed landscape-level land-cover-type richness effects on landscape-level functioning including satellite-sensed primary productivity, phenology and land-surface albedo across large environmental gradients. Study plots contained real-world landscapes and were selected according to the structure of designed biodiversity experiments.
Research sample	We sampled landscape plots covering either 6.25 or 25 ha and containing 1 to 4 different land-cover types across the whole country of Switzerland. These landscape plots covered six different biogeographic regions and an altitude range of 193-3,279 m above sea level. The land-cover types contained in these landscape plots were: forest, grassland, arable, unproductive vegetation, urban, bare land and water.
Sampling strategy	Within each block (biogeographic region x altitude-range combination) of our study, we selected plots that represented gradients in land-cover type richness and contained as many different land-cover type compositions as possible, each replicated up to 24 times. Further criteria were (1) that plots were sufficiently distant from each other, (2) altitude and land-cover type fraction were near-orthogonal with land-cover type richness, and (3) all land-cover types were present in the plots with similar area.
Data collection	Landscape functioning measures were derived from satellite-sensed (MODIS instrument) vegetation activity and albedo data, at a spatial resolution of 250 m (product name: MOD13Q1) and 500 m (product names: MOD13A1 and MCD43A3). Land-cover type data was derived from point data (100 m spatial resolution) provided by the Swiss Federal Statistical Office (GEOSTAT, product name: NOAS04) and aggregated at the scale of the MODIS data. Topographic data was derived from a digital elevation model (product name: DHM25) with 25 m spatial resolution provided by the Swiss Federal Office of Topography (swisstopo). Climate related data was derived from interpolated gridded monthly datasets with a spatial resolution of 0.0208 decimal degree provided by the Swiss Office of Meteorology and Climatology (MeteoSwiss, product names: TabsM, RhiresM, msg.SIS.M).
Timing and spatial scale	Our study covered 41,248 km^2 and 17 years (MODIS data for the years 2000-2016). Land-cover type data (see above) is based on data collected from 2004-2009. The topographic data was collected by swisstopo in the years 1978-2001. Climate data refers to the years 2000-2014 (temperature and precipitation), and 2004-2014 (irradiation).
Data exclusions	We did not exclude any data from our datasets and statistical analyses.
Reproducibility	All data processing and analyses are implemented in R (version 3.5; http://r-project.org) and can be reproduced easily.
Randomization	All plots were selected randomly, following criteria independent of landscape functioning.
Blinding	Does not apply to our study.

Did the study involve field work? ☐ Yes ☒ No

Reporting for specific materials, systems and methods

We require information from authors about some types of materials, experimental systems and methods used in many studies. Here, indicate whether each material, system or method listed is relevant to your study. If you are not sure if a list item applies to your research, read the appropriate section before selecting a response.

Materials & experimental systems

n/a	Involved in the study	
☒	☐	Antibodies
☒	☐	Eukaryotic cell lines
☒	☐	Palaeontology
☒	☐	Animals and other organisms
☒	☐	Human research participants
☒	☐	Clinical data

Methods

n/a	Involved in the study	
☒	☐	ChIP-seq
☒	☐	Flow cytometry
☒	☐	MRI-based neuroimaging
