# Supplementary material for: Terrestrial land-cover type richness is positively linked to landscape-level functioning
Source: Nat Commun. 2020 Jan 9;11:154. doi: 10.1038/s41467-019-14002-7 (PMC6952349; doi:10.1038/s41467-019-14002-7)
Supplement: Supplementary file 2 — Peer Review File [file 41467_2019_14002_MOESM2_ESM.pdf]

Reviewers' comments:

Reviewer #1 (Remarks to the Author):

NCOMMS-18-31692

Terrestrial landscape diversity promotes landscape-level functioning

by Oehri et al.

I have read through this paper and found it potentially important as it has a focus on the scale that would be relevant to “the real-world”. I agree with their notion that knowledge gained from this kind of studies would be important to the real-world settings in theory and most importantly in practice. I like their challenge in shedding light on large-scale issues and aiming to inform policy. However, I have substantial concerns mostly in their interpretations (actually, speculations). It is indeed unfortunate that they did not focus on what they found. They speculated untested mechanisms and processes. They did not quantify any biological processes such as dispersal, beta-diversity, species asynchrony and so on; nevertheless, they mostly discussed ecological processes that can be observed in local-scale biodiversity experiments and theories. Also, I am not comfortable to see that land-cover richness as a surrogate of beta-diversity. This over-simplification seriously undermines the past important works and discussion for beta-diversity. Furthermore, they did not provide some of important information such as the validation using the monitoring data, which was only described in the discussion. Overall, I like their approach, but I am not comfortable to see how the story is framed without any evidence.

L35-36 and 44: I disagree with this oversimplification for beta-diversity.

L61: Better to cite more general syntheses.

L63: Information is too old.

L72: They are theoretical work. In this context, it would be better to cite empirical studies as well.

L70-77: These explanations for ecological systems based on species interactions are informative but they are not well fit with the present study, which has no data to test these mechanisms.

L77-78: Needs more explanations for “vegetation-atmosphere interactions”. It is fairly unclear.

L87: I have a major concern. Land-over type richness also includes urban areas. I have no idea how including urban areas can increase vegetation productivity. I found no rationale to consider urban and semi-urban areas as equivalent to forest and other types of natural vegetation.

L98-100: Needs references to support these notions.

L177-178: I do not understand how the present study is linked with the concept of meta-ecosystems. No connections, flows and interlinkages between ecosystems in terms of species immigration/emigration or energy flows are tested here. Some of arguments the authors rely on are based upon theoretical works for meta-community and meta-ecosystem. However, the present results are not for describing these ecological mechanisms at all.

L187-189: This is a typical example. No rationale is provided for this speculation.

L197-199: Same as the above. The study does not have any information to discuss these biological mechanisms.

L200: I am not convinced for the definition of beta-diversity here.

L203: “species, see the above.

L204-205: This is again merely a speculation.

L217: This methodological approach is not described in the methods and elsewhere. So the meaning of “very same” is unclear.

L222-231: Again, these sentences are merely speculations. The study has no evidence to discuss them.

L233-235: There is no rationale to support this potential mechanism. Really, speculative.

L279-280: Again, I disagree with this over-simplification.

L304: Agree. But the authors failed to focus on their key findings.

L308-311: Overall, I am not convinced by this study, and thus my impression Is that this notion cannot be warranted.

L319: It is unclear to me how these scales are warranted for the present study, merely beyond the reason of data structure. Also, it would be great to analyze at larger scales.

L342-343: Again, I have no idea how meaningful to increase richness by addition urban area.

L344-345: No explanation is provided to justify this. Needs explanations.

Figures: Needs better work. Most texts are too small and resolution is not great. Also, some explanations are lacing from the captions, for instance, VIS is not explained in the caption of Fig. 3.

Overall, I like the approach of the study but I am not comfortable the way how the authors frame their study and interpret their results. I strongly suggest the authors to reframe the study and primarily focus on what they found rather than speculating based on the existing knowledge from experiments.

Reviewer #2 (Remarks to the Author):

In this paper, Oehri et al. combined land cover survey and remote sensing data to investigate the relationship between landscape diversity and landscape functioning. In order to control the potential confounding effects from environmental factors, the authors defined and performing their analyses within blocks which characterize different biogeographic regions and altitudes. Their analyses showed that both mean and stability of landscape productivity, growing season length, and land surface albedo increased with landscape diversity, as measured by the number of land-cover types. The findings are interesting and important, as it extends previous BEF research to the scale more relevant to ecosystem management. The paper is well written and clear. However, I have a few comments to be considered before publishing.

The authors have interpreted the landscape diversity as beta diversity. But usually beta diversity measurs the spatial turnover across pre-defined “local” plots, and here it is unclear what are the “local” plots. Indeed, to me this metric looks more like gamma diversity. Anyway I suggest to better

justify this terminology (LD as beta diversity), although the authors' explanations based on spatial asynchrony/insurance still apply in either case.

Besides, there are recently several papers to examine the effect of beta diversity and landscape heterogeneity on ecosystem processes, e.g. McGranahan et al. (2016), Burley et al. (2016), Hautier et al. (2018), etc. They should be acknowledged. In doing so, it is important to highlight the novelty of the current study beyond previous ones, including the authors' own recent paper Oehri et al. (2017).

To control for the confounding effects from environmental variables, the authors have defined blocks in which species lineages and environment should be more similar. This is appreciated. However, a block still covers a considerably large spatial extent, thus environmental variables may exhibit significant variations. Have you examined the correlation between LD & LF and environmental variables (e.g. temperature, precipitation, etc.), to ensure that the correlation between LD and LF is not, at least not entirely, explained by environmental factors?

One conclusion emerging from the Discussion is that, the effect of LD on LF is more linear and thus different from previous findings that biodiversity effect saturated at high diversity. Maybe I missed something, but isn't this simply because you have used a linear model? If you want to emphasize this point, I do think that a statistical comparison between your linear model and an alternative nonlinear model (e.g. a saturated curve) is required.

Lastly, I have a few questions and suggestions to improve the clarity of Methods.

(1) On phenology. The smoothing approach as mentioned in Fig. 1b was not explained in the Methods. How is the curve generated? In addition, Fig. 1b is quite similar to the Figure 2 of your PNAS paper, so I suggested to move it into appendix.

(2) Definition of overyielding. The authors have calculated a simple average across land-cover types, but should a weighted average be used (weighting by the proportion of each land-cover type)? In addition, figure 3 showed how the overyielding changes with LD, but how does the original value (of EVI, GSL, etc.) change with LD?

(3) Effect size. The calculation of Zr should be clarified.

Minor comments:

L103: Unclear sentence. How is "average land-cover type composition" defined?

L203-205: Confused. Why the effect of beta diversity can be through altering alpha diversity?

L376: the definition of CV - as the ratio of standard deviation to mean - should be clarified.

Reference:

Burley, H. M., Mokany, K., Ferrier, S., Laffan, S. W., Williams, K. J., & Harwood, T. D. (2016). Primary productivity is weakly related to floristic alpha and beta diversity across Australia. Global Ecology and Biogeography, 25(11), 1294-1307.

Hautier, Y., Isbell, F., Borer, E. T., Seabloom, E. W., Harpole, W. S., Lind, E. M., ... & Bakker, J. D. (2018). Local loss and spatial homogenization of plant diversity reduce ecosystem multifunctionality. Nature ecology & evolution, 2(1), 50.

McGranahan, D. A., Hovick, T., Elmore, R. D., Engle, D. M., Fuhlendorf, S. D., Winter, S. L., ... & Debinski, D. M. (2016). Temporal variability in aboveground plant biomass decreases as spatial variability increases. Ecology.

Oehri, J., Schmid, B., Schaepman-Strub, G., & Niklaus, P. A. (2017). Biodiversity promotes primary productivity and growing season lengthening at the landscape scale. Proceedings of the National Academy of Sciences, 114(38), 10160-10165.

Reviewer #3 (Remarks to the Author):

Overall this is a well-conceived and well-written paper addressing an important issue in ecology - the relationship between biodiversity and ecosystem functioning. What makes this paper unique is the focus on landscape level 'diversity' and functioning. This perspective is important if we are to understand the importance of biodiversity in a 'real world context' of which there have been recent calls and growing recognition of the potential for mismatches between BEF experiments and real-world effects. While the paper has many strengths, I believe the clarity of its overall message could be improved. Below I provide more detailed comments that the authors should address.

Lines 51-52 – this statement could be clearer – consider revising to ‘the incremental benefits of adding species diminish substantially with increasing richness’ (i.e., benefits of adding species most pronounced at low richness levels)

Line 56 – I would say ‘species-level richness’

Line 57 – replace ‘which’ with ‘what’

Line 58 – what about citing Duffy et al. 2017 paper?

Line 62 – replace ‘By today’ with ‘Currently’

Line 65 – are they always ‘strongly interconnected’? don’t human activities often modify or diminish these interconnections (whatever they may be)?

Line 71 – beta diversity is used in many ways – I would say that the way you define it is the less often used way of using this metric – need to be clear about how yours differs from many other studies comparing composition of intact communities rather than types of landscape units

Lines 76, 207 – decorrelation is a weird term – why not just say a lack of correlation

Line 94-95 revise sentence – consider revising to ‘’is the confounding effects of correlations between multiple driving factors’

Lines 84-87, 342 – Given that you measured land cover richness, you should refer to this metric as “landscape richness”, LR; you did not measure ‘spatial dissimilarities among ecosystems’ this would be a more like a dissimilarity (beta-diversity metric). This raises the question though, why do you focus on beta-diversity in the Introduction, when ultimately you only calculate richness of landscape elements? It seems like a more quantitative measure of diversity would be to calculate diversity incorporating abundances of each landscape element in a fixed area. By using richness, you ignore the potential for sampling effects, i.e., a particular landscape element having large effects on meta-ecosystem function (this is a problem with many BEF studies that use richness).

Lines 171-174 – I found this result weak – seems like you are comparing apples and oranges here.

Line 180 – what do you mean by ‘even abundance in mixture’? does this mean that each cover type covered the same area? How did you determine this?

Line 186-188 – not sure how you can say this given that you use such coarse land cover elements

Line 197-199 – again not sure how you can conclude this given that you do not assess asynchrony of land cover elements

Line 200 – how can you refer to landscape diversity as beta-diversity if you did not measure this? Need to be very clear that what you measured is only richness of landscape elements

Lines 211-231 – I don’t necessarily buy this analysis. It is unclear to me how much abundance of landscape elements in an area would match to richness of shrubs/herbaceous species. Also, are the plots arrayed across all landscape elements included in your analysis. Unless you can provide more detail here, I would not include this analysis.

Lines 232-275 – what about sampling effects? Do you tend to see higher LF when certain landscape elements are included?

Line 276 – revise to ‘compositional diversity is intrinsically linked to’

Lines 311-313 – I think you have weak evidence for this assertion

Reviewer #1 (Remarks to the Author):

NCOMMS-18-31692
Terrestrial landscape diversity promotes landscape-level functioning
by Oehri et al.

I have read through this paper and found it potentially important as it has a focus on the scale that would be relevant to "the real-world". I agree with their notion that knowledge gained from this kind of studies would be important to the real-world settings in theory and most importantly in practice. I like their challenge in shedding light on large-scale issues and aiming to inform policy.

However, I have substantial concerns mostly in their interpretations (actually, speculations). It is indeed unfortunate that they did not focus on what they found. They speculated untested mechanisms and processes. They did not quantify any biological processes such as dispersal, beta-diversity, species asynchrony and so on; nevertheless, they mostly discussed ecological processes that can be observed in local-scale biodiversity experiments and theories.
We fully agree with this point and have now re-framed our Introduction and Discussion. We now focus on our core analysis, which is about effects of land cover-type richness (in short landscape richness, LR) on landscape functioning (LF). As we now explain in detail, this corresponds to an organizational shift from ecosystems to landscapes (e.g. lines 67-69, 93-104 and 172-174). Our LR measure is related to the diversity of ecosystem types present in a landscape.
Given that LR may be correlated with local species richness and that effects of the two on LF therefore could be confounded, we added a supplementary analysis (Supplementary Analysis 2) in which we explore this possibility. This analysis showed that in our data set LR and species richness are largely independent of each other, suggesting that the reported LR effects on LF variables were underpinned by mechanisms unrelated to species richness. We now have added sections to both Introduction (e.g. lines lines 67 – 92) and Discussion (e.g. lines 196-212) where we speculate about the potential nature of such mechanisms and indicate how they could be analyzed.

Also, I am not comfortable to see that land-cover richness as a surrogate of beta-diversity. This over-simplification seriously undermines the past important works and discussion for beta-diversity.
We fully agree with this point and have re-framed our work to now focus on what was our primary interest, namely the effects of land-cover type richness (LR) on landscape-scale functioning (LF).

Furthermore, they did not provide some of important information such as the validation using the monitoring data, which was only described in the discussion.
We now focus on LR as explanatory variable, but point out the relevance of the question to which extent LR is correlated with local species richness. As mentioned above we have tested this in Supplementary Analysis 2 and found that LR was largely independent of species richness.
L63: Information is too old.
We now refer to the importance our study "when considering today's terrestrial area which is profoundly shaped by anthropogenic activities" in the Discussion on lines 211-212 and refer to Ellis et al. 2010.

L72: They are theoretical work. In this context, it would be better to cite empirical studies as well.
L70-77: These explanations for ecological systems based on species interactions are informative but they are not well fit with the present study, which has no data to test these mechanisms.
L77-78: Needs more explanations for “vegetation-atmosphere interactions”. It is fairly unclear.
With the explicit focus on LR, this section has become obsolete. Instead, we now provide examples and discuss the importance of large-scale interactions among land-cover types (LC types), which are the mechanisms that are related to our focus on larger entities, namely land-cover units (LC units; e.g. Introduction lines 67 – 92 and Discussion lines 196-212).
Empirical studies or effects of β- and γ-diversity on LF variables are referred to in the Discussion (e.g. lines 181-196).

L87: I have a major concern. Land-over type richness also includes urban areas. I have no idea how including urban areas can increase vegetation productivity. I found no rationale to consider urban and semi-urban areas as equivalent to forest and other types of natural vegetation.
This is indeed a very interesting point! As we now explain in the Introduction (e.g. lines 83-92) and the Discussion (e.g. lines 196-204), shifting the focus from species to LC types allows us to include LC types in which vegetation is less important (in the extreme case even absent). LC units of this LC type will interact with units of other LC types for example through heat exchange. An urban area might heat up more quickly than its vegetated surrounding and this heat can then be transferred into vegetated adjacent areas through advection where it can modify growing season length and primary productivity. We discuss a range of further mechanisms along these lines (e.g. lines 204-208). Such effects cannot be described in terms of species richness. As we now discuss in the revised manuscript, using, where available, both LR and species richness as explanatory variables of LF may thus capture effects that are driven by different mechanisms (lines 208-2012).

L98-100: Needs references to support these notions.
Floristic analyses led to the identification of these distinct biogeographic regions. We have added a corresponding reference (Wohlgemuth 1996; lines 112, 1116, 306). The regions also differ with respect to geology. For example, the Jura region has limestone parent material, whereas the Alpine regions have siliceous parent material, as we now mention in the Methods section (line 306). These regions are clearly demarcated and recognized by the Swiss Federal Office for the Environment. Altitude, the second blocking dimension, was used because it is strongly related to climate, in particular temperature.
In more general terms, blocks are used in experimental research as precautionary measure to capture spatial variation even if the specific nature of this variation is unknown. In our study, we therefore used these blocks to avoid accidental correlation between LR and unknown spatial variation, which could have happened with a completely randomized design.

L177-178: I do not understand how the present study is linked with the concept of meta-ecosystems. No connections, flows and interlinkages between ecosystems in terms of species immigration/emigration or energy flows are tested here. Some of arguments the authors rely on are based upon theoretical works for meta-community and meta-ecosystem. However, the present results are not for describing these ecological mechanisms at all.

We have re-framed our work, now focusing on LR as explanatory variable. Our approach is phenomenological and focused on the statistical relationship between LR and LF, analogous to so-called BEF field experiments, which focus on the relationship between species richness (biodiversity, B) and ecosystem functioning (EF). Even though we did not study fluxes of material, energy, or organisms between LC units, it is evident that interactions between these must underlie the observed positive LRLF effects. Such interactions are emergent properties absent within a single LC unit (or isolated ecosystem). We now use the term "meta-ecosystem" only in reference to other work, because there the term often refers to systems of LC units (ecosystems) belonging to the same LC type (ecosystem type) whereas in our analysis we are more concerned with interactions between LC units belonging to different LC types. We now acknowledge that we did not study particular biological mechanisms of interaction often addressed in work on meta-ecosystems (e.g. lines 100-104, 213-217) and thus have limited our references to meta-ecosystems (line 74).

L187-189: This is a typical example. No rationale is provided for this speculation.
L197-199: Same as the above. The study does not have any information to discuss these biological mechanisms.
L200: I am not convinced for the definition of beta-diversity here.
L203: "species, see the above.
L204-205: This is again merely a speculation.
We agree, this section has been removed.

L217: This methodological approach is not described in the methods and elsewhere. So the meaning of "very same" is unclear.
We now have added the Supplementary Analysis 2 that provides methods and results in detail.

L222-231: Again, these sentences are merely speculations. The study has no evidence to discuss them.
L233-235: There is no rationale to support this potential mechanism. Really, speculative.
L279-280: Again, I disagree with this over-simplification.
We agree, this section has been removed.

L304: Agree. But the authors failed to focus on their key findings.
In the revised text, we now focus on the core data which we have collected and the statistical analysis of these.

L308-311: Overall, I am not convinced by this study, and thus my impression Is that this notion cannot be warranted.
We agree that our discussion of particular mechanisms was overly speculative. In the revised text, we now focus on the statistical LRLF effects that we found. As stated above, in the course of the revision we have repeated the entire analysis with two completely new sets of landscape plots and could confirm the previous results. We thus are convinced that the observed LRLF relationships are statistically robust but point out that these are phenomenological results, analogous to BEF relationships reported in other biodiversity research (e.g. lines 100-104)

L319: It is unclear to me how these scales are warranted for the present study, merely beyond the reason of data structure. Also, it would be great to analyze at larger scales.
We have largely toned down the notion of scales. We focused on 250x250 m and 500x500 m landscape plots because satellite-sensed data were available at these scales. We also

considered analyzing larger spatial scales. However, this proved impossible because landscapes in Switzerland are so fragmented that almost no areas of larger size are of a single land-cover type. In fact, the data set with 500x500m plots already was much smaller for this very reason. However, we think the fact that we found very similar effect sizes in both 250x250m and 500x500m data sets, both in the original analysis and now again with a new set of landscape plots, underlines the consistency of the observed LRLF relationships.

L342-343: Again, I have no idea how meaningful to increase richness by addition urban area.
See our explanation above why this LC type can contribute to landscape-level diversity effects.

L344-345: No explanation is provided to justify this. Needs explanations.
Indeed this was not well explained. We now explain in detail how we aggregated the original land-cover information into the eight broad classes that we used (lines 331-341).

Figures: Needs better work. Most texts are too small and resolution is not great. Also, some explanations are lacing from the captions, for instance, VIS is not explained in the caption of Fig. 3.
All figures have been re-designed, focusing on clarity.

Overall, I like the approach of the study but I am not comfortable the way how the authors frame their study and interpret their results. I strongly suggest the authors to reframe the study and primarily focus on what they found rather than speculating based on the existing knowledge from experiments.
In the revision, we now focus on the primary data and have conceptually re-framed our study, focusing on LR instead of beta diversity. We think that this addressed the indeed very important points raised by reviewer 1.

Reviewer #2 (Remarks to the Author):

In this paper, Oehri et al. combined land cover survey and remote sensing data to investigate the relationship between landscape diversity and landscape functioning. In order to control the potential confounding effects from environmental factors, the authors defined and performing their analyses within blocks which characterize different biogeographic regions and altitudes. Their analyses showed that both mean and stability of landscape productivity, growing season length, and land surface albedo increased with landscape diversity, as measured by the number of land-cover types. The findings are interesting and important, as it extends previous BEF research to the scale more relevant to ecosystem management. The paper is well written and clear. However, I have a few comments to be considered before publishing.

The authors have interpreted the landscape diversity as beta diversity. But usually beta diversity measures the spatial turnover across pre-defined "local" plots, and here it is unclear what are the "local" plots. Indeed, to me this metric looks more like gamma diversity. Anyway I suggest to better justify this terminology (LD as beta diversity), although the authors' explanations based on spatial asynchrony/insurance still apply in either case.
As explained in our answers to the first reviewer, we recognized that our analysis was ill-framed by focusing on beta-diversity. We now focus on the richness of land-cover types (LR; e.g. lines 93-104 and 172-174), which we think is appropriate and novel.

Besides, there are recently several papers to examine the effect of beta diversity and landscape heterogeneity on ecosystem processes, e.g. McGranahan et al. (2016), Burley et al. (2016), Hautier et al. (2018), etc. They should be acknowledged. In doing so, it is important to highlight the novelty of the current study beyond previous ones, including the authors' own recent paper Oehri et al. (2017).
Thank you for pointing out these references. We now focus on LR and discuss its relation to beta and gamma diversity and findings from these studies (lines 186-192). We highlight the novelty of the current approach compared to these previous studies, including our own (e.g. lines 67-76 and 196-212).

To control for the confounding effects from environmental variables, the authors have defined blocks in which species lineages and environment should be more similar. This is appreciated. However, a block still covers a considerably large spatial extent, thus environmental variables may exhibit significant variations. Have you examined the correlation between LD & LF and environmental variables (e.g. temperature, precipitation, etc.), to ensure that the correlation between LD and LF is not, at least not entirely, explained by environmental factors?
This indeed is an important point. We have analyzed these correlations and, because we think this is such a critical point in our study, now present them as Figure 2 in the main text. It shows that the block structure we chose substantially reduces correlations with environmental variables. We tested for these and, within blocks, none of the environmental variables were significantly related to our explanatory variable LR (Fig. 2).
To make absolutely sure that any remaining confounding, even if small, would not change our findings, we adjusted for all environmental variables before testing for log(LR) (see figure below). Effect sizes remain essentially unchanged. We did not include this figure in the main text because we think Fig. 2. conveys the same information.

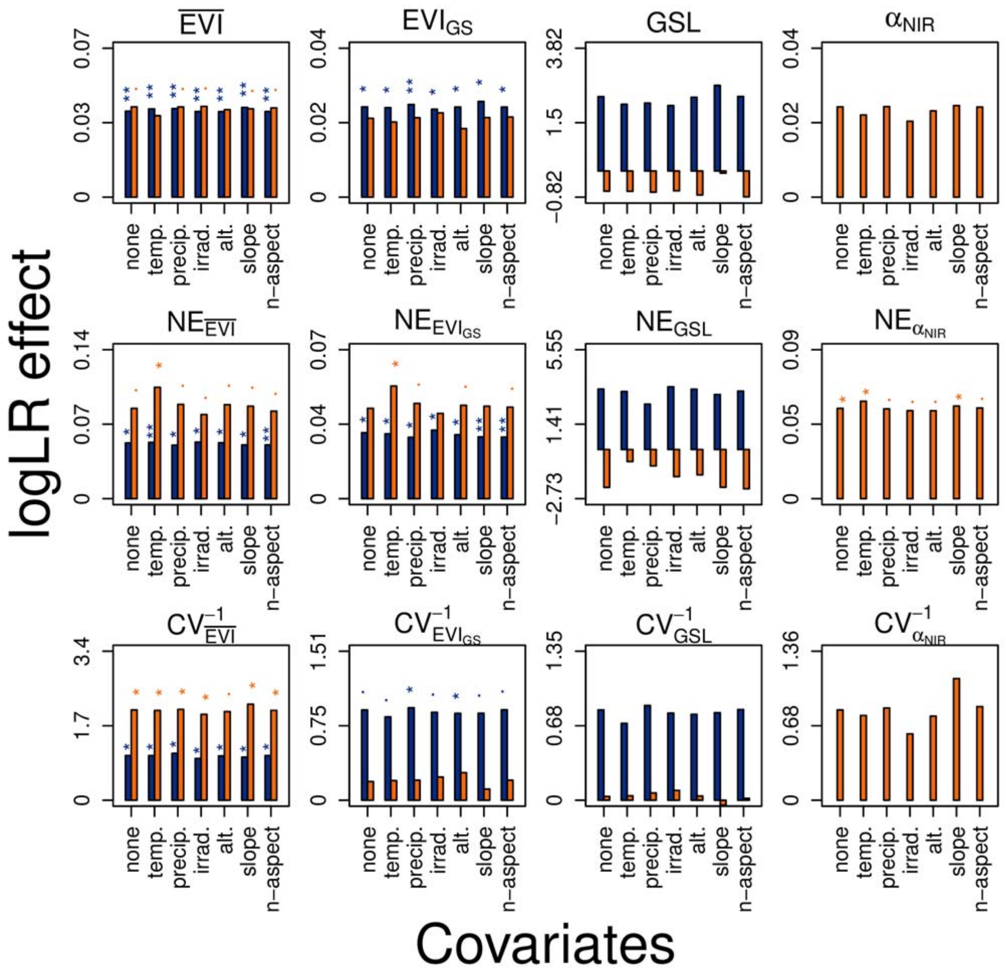

*Figure R1 | Landscape richness effects on landscape functioning variables with and without prior adjustment for environmental covariates. Bars represent estimated coefficients of log-transformed landscape richness [log(LR)]. All models are first adjusted for block. X-axis: "none": no co-variate was fitted; "temp.": mean annual temperature; "precip.": yearly precipitation; "irrad." mean annual surface incoming shortwave radiation; "alt.": altitude; "slope": slope; "n-aspect": north-south component of the aspect. $\overline{EVI}$ and EVI_{GS}: mean and integrated growing season productivity; GSL: growing season length; . α_{NIR}: near-infrared albedo; NE: net biodiversity effect for these variables; CV^{-1}: temporal stability of these variables. (. P<0.1; * P<0.05; ** P<0.01; *** P<0.001; blue: 250×250 m landscape plots, n=237; orange: 500×500 m landscape plots, n=77).*

One conclusion emerging from the Discussion is that, the effect of LD on LF is more linear and thus different from previous findings that biodiversity effect saturated at high diversity. Maybe I missed something, but isn't this simply because you have used a linear model? If you want to emphasize this point, I do think that a statistical comparison between your linear model and an alternative nonlinear model (e.g. a saturated curve) is required.
We have removed this section because the re-analysis we did with new data did not corroborate this finding. Now we have evaluated a larger set of models and found that using the logarithm of LR resulted in a better model fit than LR, increasing the similarity

with BEF studies, which also mostly find a linear relationship between the logarithm of species richness and ecosystem functioning. We thus use log(LR) throughout and explain model selection in more detail in the Methods (lines 407-411).

Lastly, I have a few questions and suggestions to improve the clarity of Methods.
(1) On phenology. The smoothing approach as mentioned in Fig. 1b was not explained in the Methods. How is the curve generated? In addition, Fig. 1b is quite similar to the Figure 2 of your PNAS paper, so I suggested to move it into appendix.
We now explain the processing of the MODIS data in detail (lines 348-355) and have removed this figure and refer to Oehri et al. 2017 instead (lines 351, 358).

(2) Definition of overyielding. The authors have calculated a simple average across land-cover types, but should a weighted average be used (weighting by the proportion of each land-cover type)? In addition, figure 3 showed how the overyielding changes with LD, but how does the original value (of EVI, GSL, etc.) change with LD?
We now use the term "net biodiversity effect" (NE) because we think it is more appropriate for terms unrelated to yield and it has been clearly defined in the citation classic of Loreau and Hector (2001). Its calculation is now based on weighted averages (weights by the proportion of each LC type in a landscape) and explained in detail in the methods section (lines 384-390).
All analyses have been conducted for both the original values and for the NE. NE results are summarized in Table 1 (instead of Figure 3 in the previous manuscript) while Figure 3 shows the original values (for which predicted LR effects have been shown in Figure 2 in the previous version of the manuscript).

(3) Effect size. The calculation of Zr should be clarified.
This information has been added (lines 427-431).

Minor comments:

L103: Unclear sentence. How is "average land-cover type composition" defined?
This wording indeed was confusing. What we meant was the fraction of a certain LC type found at a particular LR level. For example, in a block where four different LC types were used to build the diversity gradient, the average LC fraction of any LC type at any LR level was ¼. We have carefully checked our data sets to corroborate this, i.e. that no correlations existed between the fraction of any LC type and LR. Because this is critically important for our "quasi-experimental" design and analysis, this is now shown in Figure S3.

L203-205: Confused. Why the effect of beta diversity can be through altering alpha diversity?
This section was removed with our focus on LR instead of beta-diversity. A reason for our investigation of LR-species richness relationships is given on lines 241-246.

L376: the definition of CV - as the ratio of standard deviation to mean - should be clarified.
We now defined CV more clearly and explain the rationale for its use (lines 375-383).

Reference:

Burley, H. M., Mokany, K., Ferrier, S., Laffan, S. W., Williams, K. J., & Harwood, T. D. (2016). Primary productivity is weakly related to floristic alpha and beta diversity across Australia. Global Ecology and Biogeography, 25(11), 1294-1307.

Hautier, Y., Isbell, F., Borer, E. T., Seabloom, E. W., Harpole, W. S., Lind, E. M., ... & Bakker, J. D. (2018). Local loss and spatial homogenization of plant diversity reduce ecosystem multifunctionality. Nature ecology & evolution, 2(1), 50.

McGranahan, D. A., Hovick, T., Elmore, R. D., Engle, D. M., Fuhlendorf, S. D., Winter, S. L., ... & Debinski, D. M. (2016). Temporal variability in aboveground plant biomass decreases as spatial variability increases. Ecology.

Oehri, J., Schmid, B., Schaepman-Strub, G., & Niklaus, P. A. (2017). Biodiversity promotes primary productivity and growing season lengthening at the landscape scale. Proceedings of the National Academy of Sciences, 114(38), 10160-10165.

Reviewer #3 (Remarks to the Author):

Overall this is a well-conceived and well-written paper addressing an important issue in ecology - the relationship between biodiversity and ecosystem functioning. What makes this paper unique is the focus on landscape level 'diversity' and functioning. This perspective is important if we are to understand the importance of biodiversity in a 'real world context' of which there have been recent calls and growing recognition of the potential for mismatches between BEF experiments and real-world effects.

While the paper has many strengths, I believe the clarity of its overall message could be improved. Below I provide more detailed comments that the authors should address.

Lines 51-52 – this statement could be clearer – consider revising to ‘the incremental benefits of adding species diminish substantially with increasing richness’ (i.e., benefits of adding species most pronounced at low richness levels)
Revised (lines 43-44).

Line 56 – I would say ‘species-level richness’
Line 57 – replace ‘which’ with ‘what’
Revised (line 47).

Line 58 – what about citing Duffy et al. 2017 paper?
We have added this important reference (line 183).

Line 62 – replace ‘By today’ with ‘Currently’
We removed the paragraph and this revision became obsolete.

Line 65 – are they always ‘strongly interconnected’? don’t human activities often modify or diminish these interconnections (whatever they may be)?
We re-wrote this section in the course of the revision. It is true that humans also fragment landscapes. The point we make here is that ecosystems are interconnected in natural landscapes, which may give rise to diversity effects through mechanisms that do not operate in experimental BEF studies. In these, plots are homogeneous, and even if connections to their surrounding exist, they are averaged out statistically though treatment randomization (lines 47-53 and 89-92).

Line 71 – beta diversity is used in many ways – I would say that the way you define it is the less often used way of using this metric – need to be clear about how yours differs from many other studies comparing composition of intact communities rather than types of landscape units.
Agreed. As stated above, we have shifted our focus from beta-diversity to the richness of land-cover (LC) types (landscape richness, LR; e.g. lines 93-104 and 172-174), which is our main design variable.

Lines 76, 207 – decorrelation is a weird term – why not just say a lack of correlation
We removed these paragraphs and this revision became obsolete.

Line 94-95 revise sentence – consider revising to ‘’is the confounding effects of correlations between multiple driving factors’
We have revised this statement accordingly (lines 108-111).

Lines 84-87, 342 – Given that you measured land cover richness, you should refer to this

metric as “landscape richness”, LR; you did not measure ‘spatial dissimilarities among ecosystems’ this would be a more like a dissimilarity (beta-diversity metric). This raises the question though, why do you focus on beta-diversity in the Introduction, when ultimately you only calculate richness of landscape elements?
We fully agree and have revised our manuscript focusing on land-cover type richness instead of beta-diversity and use the abbreviation LR (e.g. lines 172-174).

It seems like a more quantitative measure of diversity would be to calculate diversity incorporating abundances of each landscape element in a fixed area. By using richness, you ignore the potential for sampling effects, i.e., a particular landscape element having large effects on meta-ecosystem function (this is a problem with many BEF studies that use richness).
This is indeed an important point. In our study the abundance of specific land-cover types was held constant across the LR gradient. This contrasts traditional BEF experiments, in which the evenness of species is not maintained throughout the experiment and often strong rank–abundance distributions develop over time.
Although there was no correlation between the abundance of land-cover types and LR in our study (landscape plots were selected to eliminate it), there was some deviation from perfect evenness on a plot-by-plot basis. We therefore used more quantitative measures of landscape diversity including Simpson’s diversity index of LC types weighted by the proportion of area they covered in a landscape plot. We also analyzed the data using metrics that focus on other aspects of landscape diversity such as the edge density and the connectedness of patches with the same land cover. All these analyses essentially yielded the same results as using LR. These alternative analyses are now presented in the Supplementary Analysis 4.

Lines 171-174 – I found this result weak – seems like you are comparing apples and oranges here.
Comparing studies and different response variables can be challenging. We think that meta-analytical effect sizes are a reasonable choice, in particular within the same or similar study designs, models and conceptually similar variables of investigation. This allows us to evaluate the strength of LRLF relationships for different responses and across studies. This is standard methodology in meta-analyses (e.g. cf. Rosenthal 1994), although we agree that there are limitations.

Line 180 – what do you mean by ‘even abundance in mixture’? Does this mean that each cover type covered the same area? How did you determine this?
This indeed was the case because we selected the plots according to this criterion (lines 320-326). Perfect evenness could not be achieved in each and every plot, but we selected the plots so that the correlation between LR and the fraction of land covered by each land-cover type was minimized. We tested this statistically and indeed no correlation was significant, despite the high power we had from the large number of plots (lines 326-328).

Line 186-188 – not sure how you can say this given that you use such coarse land cover elements
Line 197-199 – again not sure how you can conclude this given that you do not assess asynchrony of land cover elements
Line 200 – how can you refer to landscape diversity as beta-diversity if you did not measure this? Need to be very clear that what you measured is only richness of landscape elements
This section was removed, and we no longer focus on species beta- and gamma-diversity.

Lines 211-231 – I don't necessarily buy this analysis. It is unclear to me how much abundance of landscape elements in an area would match to richness of shrubs/herbaceous species. Also, are the plots arrayed across all landscape elements included in your analysis. Unless you can provide more detail here, I would not include this analysis.

We think that it is important to evaluate, at least as far as this is possible, whether the effect of LR we report here might 1) have been driven by indirect effects of LR on local species richness, or 2) whether LR and local species richness are correlated for other reasons so that the two are statistically confounded.

We have therefore carefully re-evaluated this additional data set and present the Supplementary Analysis 2 that shows that LR and species richness at these two scales were uncorrelated. As we discuss, this suggests that the observed LRLF relations are underpinned by mechanisms unrelated to local species richness.

Lines 232-275 – what about sampling effects? Do you tend to see higher LF when certain landscape elements are included?

What we found is that LC types differ in level of functioning. These differences also are evident from the LF values in land-cover mixtures that contain these LC types. However, a sampling effect analogous to the dominance of productive species in mixed communities in our setting would be difficult to achieve because, within the temporal scales considered, LC types with higher "performance" could not increase their dominance at the expense of LC types with lower performance. In contrast, when sampling effects occur in BEF experiments, communities of originally high richness become dominated by high-performing species, and in the most extreme case become monocultures.

We searched for evidence of "complementarity-like" effects on the interactions of LC types, i.e. whether LC-type pairs that contained a particular LC type had a systematically positive or negative interactive effect on LF. What we found is that these specific interactions — quantified in the diallel framework as "specific combining ability" (SCA) — indeed depended significantly on the particular pairing of land-cover types. However, there was no consistent pattern in SCA across blocks of functions. We report these results as Supplementary Analysis 1.

Line 276 – revise to 'compositional diversity is intrinsically linked to'

Revised (line 268).

Lines 311-313 – I think you have weak evidence for this assertion

We now provide additional evidence for this point (Supplementary Analysis 2). In particular, we were able to reproduce the same patterns with a larger set of landscape plots, and explain that for these LR was not correlated with species richness.

Reviewers' comments:

Reviewer #1 (Remarks to the Author):

NCOMMS-18-31692A

Terrestrial land-cover type richness promotes landscape level functioning

by Oehri et al.

First, I acknowledge the authors to thoroughly revise the manuscript, by carefully addressing my concerns. They now use much clearer languages and removed large parts of confusing and vague arguments. I like the present manuscript to explicitly mention their focus on land-cover type richness and distinguish it from other metrics of diversity. They are also, to some degree, more explicit in explaining how the present framework is different from local-scale biodiversity-ecosystem functioning experiments. The present results are interesting and could potentially have large implications. I also find important implications such as those for potential roles of landscape configurations (L268-) and so on. Overall, I find much novelty in the present manuscript compared to the previous one.

However, please note that I still have substantial concerns. Although the findings presented in this manuscript are fundamentally intriguing and interesting, I cannot be fully convinced. First, I am still wondering; suppose we have primary forests within a region (LR =1), would it be worse than having lots of human-modified lands (LR > 1)? In my previous assessment, I asked the authors how they can justify urban areas can be equally important to other land types in terms of vegetation productivity. They now have some speculations for this in the manuscript with the detailed explanations in their rebuttal letter. If growing season could become longer and temperature could increase because of urbanization (as explained their letter), then respiration could also increase. In such case, net primary productivity does not necessarily increase and could even decrease. I cannot be convinced by the speculation in L202- in the revised manuscript. Importantly, this and related notions could be seriously misleading, as these explanations could implicitly justify further land conversion including urbanization and fragmentation. This is a serious issue, given a potential impact of the journal on society.

I find that discussion related to local BEF studies is still over-speculated. I thank the authors to eliminate lots of uncertainties but still I feel some notions somehow undermine past and present efforts in this study domain. Productivity supported by organisms (biological processes) should be measured, if their primary interest would be related to BEF theory. However, most arguments for this study to explain productivity is not necessarily/directly related to biotic attributes (e.g, heat subsides and advective heat transport). Thus, I do not find a strong rationale for this study to have discussion that is tight asscoaietd with BEF studies. Also, although I welcome this kind of sentences to explain potential mechanisms underlying the observed findings, I find many explanations are too

speculative. By reading through the sentences in L260-263, I am quite confusing as I am not told about the consequences on NPP in these different land use types.

Overall, I like the study that has a great attempt to focus on potential linkages between large-scale properties of lands and landscape-level functioning. Given the situation of ongoing land-use change, understanding this (especially its potential mechanisms) is practically important. Thus I agree with the last notion in this manuscript (L295-297). However, I hesitate to recommend this work to be published in the journal, because of the above issues. Especially, I have a large concern for the explained, potential roles of urban areas to increase regional productivity, which could unexpectedly mislead society to discourage land conservation and to promote further land conversion and fragmentation.

Reviewer #2 (Remarks to the Author):

I appreciate the authors' careful work in revising the manuscript and addressing the comments by reviewers. This revised manuscript had clarified the conceptual framework and now focused on the relationship between landscape richness (LR) and landscape-level functioning and stability (LF), aiming to testify and extend findings from local-scale BEF studies. I still find the study well conducted and clearly written. I do not have substantial concerns, but two suggestions that may strengthen the discussion.

First, I feel that discussion on the possible reasons for the positive relation between LR and LF can be expanded, in order to make connection to the existing knowledge in the BEF field. For instance, the authors explained how urban areas may benefit neighboring patches by regulating the hydro-heat processes. Such "facilitation" effect has been considered as an important mechanism in BEF studies (e.g. Cardinale et al. 2002). Besides, the potential mechanisms for the stabilizing effect of LR was largely unexplained. One possible explanation is that different land-cover types may exhibit asynchronous responses to environmental fluctuations, thus stabilizing landscape-level dynamics (Wang & Loreau 2016).

Besides, the results (figures 3-5) showed that the relationship between LR and LF was stronger for lower grain size (25*25), compared to larger one (50*50). This pattern is consistent with recent studies examining the scale dependence of tree diversity on functioning (Chisholm et al. 2013). I suggest to discuss this point, at least briefly.

Reference:

Cardinale, B. J., Palmer, M. A., & Collins, S. L. (2002). Species diversity enhances ecosystem functioning through interspecific facilitation. Nature, 415(6870), 426.

Wang, S., & Loreau, M. (2016). Biodiversity and ecosystem stability across scales in metacommunities. Ecology letters, 19(5), 510-518.

Chisholm, R. A., Muller-Landau, H. C., Abdul Rahman, K., Bebber, D. P., Bin, Y., Bohlman, S. A., et al. (2013). Scale-dependent relationships between tree species richness and ecosystem function in forests. Journal of Ecology, 101(5), 1214-1224.

October 7, 2019

Detailed point-by-point reply to the individual comments of the reviewers

We thank the reviewers for their helpful comments. Here, we provide detailed answers to the points raised by the reviewers. To increase readability, reviewer comments are written in blue. We have highlighted all the changes to the main text in yellow in the uploaded manuscript file.

Reviewer #1 (Remarks to the Author):

Terrestrial land-cover type richness promotes landscape level functioning by Oehri et al.

First, I acknowledge the authors to thoroughly revise the manuscript, by carefully addressing my concerns. They now use much clearer languages and removed large parts of confusing and vague arguments. I like the present manuscript to explicitly mention their focus on land-cover type richness and distinguish it from other metrics of diversity. They are also, to some degree, more explicit in explaining how the present framework is different from local-scale biodiversity-ecosystem functioning experiments. The present results are interesting and could potentially have large implications. I also find important implications such as those for potential roles of landscape configurations (L268-) and so on. Overall, I find much novelty in the present manuscript compared to the previous one.

We are happy to see that our revision improved clarity, which indeed was needed.

However, please note that I still have substantial concerns. Although the findings presented in this manuscript are fundamentally intriguing and interesting, I cannot be fully convinced. First, I am still wondering; suppose we have primary forests within a region (LR =1), would it be worse than having lots of human-modified lands (LR > 1)? In my previous assessment, I asked the authors how they can justify urban areas can be equally important to other land types in terms of vegetation productivity. They now have some speculations for this in the manuscript with the detailed explanations in their rebuttal letter. If growing season could become longer and temperature could increase because of urbanization (as explained their letter), then respiration could also increase. In such case, net primary productivity does not necessarily increase and could even decrease. I cannot be convinced by the speculation in L202- in the revised manuscript. Importantly, this and related notions could be seriously misleading, as these explanations could implicitly justify further land conversion including urbanization and fragmentation. This is a serious issue, given a potential impact of the journal on society.

This indeed is a very important point. What our data and analyses show is that, on average, given a large area that contains a number of different land-cover types (e.g. 25% forest, 25% urban, 25% grassland, and 25% water), this large area will have a higher total productivity when these land-cover types are intertwined (mixed) at the scale we studied than if they remain separate.

This finding is statistically robust; in particular, we were able to reproduce it in a complete re-analysis carried out for the revision, using a larger, independent, set of plots. The effect also was reproducible at two different spatial scales.

The pattern we report corresponds exactly to the one typically found in plot-scale BEF studies: communities with multiple species *on average* achieve a higher productivity than the *average* productivity of the monocultures of these species. However, this is not the case for all the possible species combinations. In particular, overyielding rarely is transgressive, i.e. the productivity of mixtures rarely exceeds the productivity of the most productive monocultures of the species present in the mixture.

We realize that these are important aspects that were not emphasized enough in our original text. As reviewer 1 rightly points out, we have a responsibility to make sure that our findings are not misinterpreted by readers outside our own field. We therefore have added a paragraph to the discussion in which we explain this result in detail (lines 261-271):

"(…) Overall, interactions among the different land-cover types thus added up to a net positive contribution to productivity, but attributing these effects to specific land-cover combinations and mechanisms was not possible and will require further research. It is critical to understand that the higher productivity in mixed landscapes we report here is an average property and unlikely to apply to all land-cover combinations individually. These "overyielding" effects may rarely be transgressive, in particular when functioning levels differ significantly between interacting land-cover types. For example, fragmenting a large continuous forest by urbanization will almost certainly reduce landscape productivity despite increased landscape richness (but the productivity of this intertwined landscape may exceed the productivity of a landscape in which the same total forest and urban areas are spatially separated)."

I find that discussion related to local BEF studies is still over-speculated. I thank the authors to eliminate lots of uncertainties but still I feel some notions somehow undermine past and present efforts in this study domain. Productivity supported by organisms (biological processes) should be measured, if their primary interest would be related to BEF theory. However, most arguments for this study to explain productivity is not necessarily/directly related to biotic attributes (e.g, heat subsides and advective heat transport). Thus, I do not find a strong rationale for this study to have discussion that is tight associated with BEF studies. Also, although I welcome this kind of sentences to explain potential mechanisms underlying the observed findings, I find many explanations are too speculative. By reading through the sentences in L260-263, I am quite confusing as I am not told about the consequences on NPP in these different land use types.

We are aware of this and therefore explicitly declare our study as phenomenological, focusing on "response patterns". We have revised our manuscript in several places to emphasize this further (e.g. lines 206-210 and lines 295-298).

However, given that this is a novel field, we think that we need to elaborate on some candidate mechanisms that may underlie the effects we report. Some of the effects we discuss are well-known from research in the domain of landscape ecology and related fields, but have not been considered in the context of the novel landscape diversity–functioning research.

Further, the analogies between response patterns to diversity at the landscape-level and at the plot-level are striking. Different spatial scales aside, the mechanisms are in some fundamental way comparable. For example, reviewer 2 highlights that spatial heat and nutrient subsidies correspond to what is referred to as facilitation in plot-level plant diversity research. Land-cover types also have characteristic traits (for example, grassland and forest differ in surface [canopy] structure and spectral properties) that affect their interactions with the environment, and, directly or indirectly, with other land-cover units. We have revised the corresponding section and outline our motivation to draw these analogies (lines 217-231):

"(…) Interestingly, some of these effects may be described in analogy to mechanisms that support biodiversity effects in plant communities. For example, beneficial climatic effects of neighboring land-cover units may be thought of as facilitation[48]. Further, land-cover units differ in qualities such as

albedo or surface roughness, properties that can be considered as functional traits and which may be useful in predicting landscape-level functioning. Also, different land-cover types may respond asynchronously to environmental drivers, thereby temporally stabilizing landscape level functioning in a way comparable to what has been found in species-level field studies and modelling analyses of communities and meta-communities[49,50]*. Overall, we therefore argue that diversity–functioning analyses at the local and landscape scale are driven by different though conceptually comparable mechanisms and provide complementary insights, and that at larger scale novel, species-independent mechanisms may play an important role. A multi-scale perspective that allows for such emergent effects appears particularly important when considering today's terrestrial land surface which is profoundly shaped by anthropogenic activities*[51]*.*

Many plant interactions ultimately are tied to physical processes, even though they often are not described in this way. For example, facilitation by nurse plants is often related to microclimatic effects (which involve heat and water budgets). Nutrient competition by root systems is ultimately mediated by diffusion processes and mass flow (i.e. advective transport). Conversely, for the vegetated land-cover types, the properties of land-cover units that determine the physical processes we discuss are biotically determined (the surface structure and albedo of a forest and grassland, to give an example).

In summary, we clearly acknowledge the differences between the scales (and elaborate on this in several places, e.g. lines 200-201 and lines 225-229), but at the same time some differences between the two scales may be less fundamental than they appear at first glance.

Overall, I like the study that has a great attempt to focus on potential linkages between large-scale properties of lands and landscape-level functioning. Given the situation of ongoing land-use change, understanding this (especially its potential mechanisms) is practically important. Thus I agree with the last notion in this manuscript (L295-297). However, I hesitate to recommend this work to be published in the journal, because of the above issues. Especially, I have a large concern for the explained, potential roles of urban areas to increase regional productivity, which could unexpectedly mislead society to discourage land conservation and to promote further land conversion and fragmentation.

Thank you for raising this very important point. In the revised manuscript, we addressed it explicitly, using the illustrative example provided here (lines 263-271). We think that this eliminates the possibility to misinterpret or abuse our findings to promote forest destruction.

Reviewer #2 (Remarks to the Author):

I appreciate the authors' careful work in revising the manuscript and addressing the comments by reviewers. This revised manuscript had clarified the conceptual framework and now focused on the relationship between landscape richness (LR) and landscape-level functioning and stability (LF), aiming to testify and extend findings from local-scale BEF studies. I still find the study well conducted and clearly written. I do not have substantial concerns, but two suggestions that may strengthen the discussion.

First, I feel that discussion on the possible reasons for the positive relation between LR and LF can be expanded, in order to make connection to the existing knowledge in the BEF field. For instance, the authors explained how urban areas may benefit neighboring patches by regulating the hydro-heat processes. Such "facilitation" effect has been considered as an important mechanism in BEF studies (e.g. Cardinale et al. 2002). Besides, the potential mechanisms for the stabilizing effect of LR was largely unexplained. One possible explanation is that different land-cover types may exhibit asynchronous responses to environmental fluctuations, thus stabilizing landscape-level dynamics (Wang & Loreau 2016).

Thank you for these helpful suggestions. We now have added a paragraph in which we draw and discuss these conceptual parallels and refer to these studies (lines 217-231).

Besides, the results (figures 3-5) showed that the relationship between LR and LF was stronger for lower grain size (25*25), compared to larger one (50*50). This pattern is consistent with recent studies examining the scale dependence of tree diversity on functioning (Chisholm et al. 2013). I suggest to discuss this point, at least briefly.

Indeed this is an intriguing pattern! We have considered this possibility earlier on but decided not to emphasize it in the manuscript, for two reasons: first, the difference between the scales is not statistically significant; second, the larger landscape plots have lower replication, and there are fewer and different land-cover combinations in this data set because many combinations simply do not occur with sufficient replication in the study area. Even if statistically significant, it would therefore not be possible to unequivocally separate effects of the land cover set from effects of scale.

Reference:
Cardinale, B. J., Palmer, M. A., & Collins, S. L. (2002). Species diversity enhances ecosystem functioning through interspecific facilitation. Nature, 415(6870), 426.
Wang, S., & Loreau, M. (2016). Biodiversity and ecosystem stability across scales in metacommunities. Ecology letters, 19(5), 510-518.
Chisholm, R. A., Muller-Landau, H. C., Abdul Rahman, K., Bebber, D. P., Bin, Y., Bohlman, S. A., et al. (2013). Scale-dependent relationships between tree species richness and ecosystem function in forests. Journal of Ecology, 101(5), 1214-1224.

Thank you for these additional references. We have included them in the revision except Chisholm et al. (see above for reasons).

REVIEWERS' COMMENTS:

Reviewer #1 (Remarks to the Author):

Thank you so much for responding to my concerns and carefully addressing the issues. As I said to the Editor, the study givens an important advancement by giving a new insight into studies of biodiversity-ecosystem functioning. The effects at large scale such as ecosystem-level diversity are largely lacking so far. And thus the study is novel. The response for being careful for urbanization is mostly satisfactory. However, I would ask the authors to further add how the study is novel by shedding a light on ecosystem-level diversity, which is one of the key facets under the CBD framework, and also giving an explicit statement for uncertainty for the land-use effects. Also, I found that there are some jargon such as overyielding, which would be unfamiliar for most audience. Please again be careful for being friendly to a wide range of audience.

Akira Mori, PhD

November 18, 2019

Point-by-point response to the comments of the referees

We thank the referees for their very useful comments throughout the revision process. Especially, we thank the referees for their effort to repeatedly scrutinize our work very carefully and for repeatedly providing very insightful and constructive critique. We think that we and the quality of our manuscript could highly profit from this process. Below, we provide detailed answers to the points raised by the referees. To increase readability, our answers are written in blue.

REVIEWERS' COMMENTS:

Reviewer #1 (Remarks to the Author):

Thank you so much for responding to my concerns and carefully addressing the issues. As I said to the Editor, the study givens an important advancement by giving a new insight into studies of biodiversity-ecosystem functioning. The effects at large scale such as ecosystem-level diversity are largely lacking so far. And thus the study is novel. The response for being careful for urbanization is mostly satisfactory. However, I would ask the authors to further add how the study is novel by shedding a light on ecosystem-level diversity, which is one of the key facets under the CBD framework, and also giving an explicit statement for uncertainty for the land-use effects. Also, I found that there are some jargon such as overyielding, which would be unfamiliar for most audience. Please again be careful for being friendly to a wide range of audience.

Akira Mori, PhD

Thank you for the positive evaluation. We address the remaining points raised as follows:

1. Indeed, the novelty of our work is the focus on entire ecosystems. We highlight this in several places. For example:
   Introduction: *"An alternative perspective, which is the one we introduce here, is that diversity effects could also occur with respect to the richness of larger organizational units, namely of entire land-cover type units, in brief "land-cover units". Land-cover type richness, in brief "landscape richness", encompasses mostly differences among ecosystems but also includes largely abiotic land-cover types such as urban areas."*
   Discussion: *"Expanding concepts of classical BEF experiments, we focused on larger scales of space and ecological organization and used the α-diversity of land-cover types in landscapes as measure of landscape diversity. This metric essentially captures the diversity of ecosystem types."*

2. We have added further statements that what we provide is phenomenological evidence, and that the specific mechanisms require future investigations. This is also reflected with the title change that no longer makes statements about causality.

3. We have shifted the emphasis to the term "net diversity effects". The term overyielding is extensively used in the BEF literature and therefore, we feel that it nevertheless deserves using. In the revised manuscript, we now use the term overyielding only three times, and always provide the context that explains its meaning:

- Results: *"The net diversity effect (NE) was positive for both $\overline{EVI}$ and EVI_{GS} (P=0.001–0.003 depending on landscape size; Table 1), i.e. both $\overline{EVI}$ and EVI_{GS} "overyielded" in mixed landscapes relative to the average of the corresponding single land-cover type landscapes."*
- Discussion: *"It is critical to understand that the higher productivity in mixed landscapes we report here is an average property and unlikely to apply to all land-cover combinations individually. These "overyielding" effects …".* Here, the context provides the explanation.
- Methods: *"For productivity terms, NE* [net diversity effect, defined in previous sentence] *often also is referred to as 'overyielding'."*
